# Alpha conotoxin-BuIA globular isomer is a competitive antagonist for oleoyl-L-alpha-lysophosphatidic acid binding to LPAR6; A molecular dynamics study

**DOI:** 10.1371/journal.pone.0189154

**Published:** 2017-12-06

**Authors:** Saima Younis, Sajid Rashid

**Affiliations:** National Center for Bioinformatics, Quaid-i-Azam University, Islamabad, Pakistan; Wake Forest University, UNITED STATES

## Abstract

Lysophosphatidic acid receptor 6 (LPAR6) is a G-protein coupled receptor (GPCR) involved in hair development and cytoskeleton formation in mammals. Its proliferation is implicated in several forms of cancer including liver cancer, squamous cell carcinoma and metastatic prostate cancer. Current study emphasizes the isolation of competitive non-lipid and stable peptide antagonists for Lysophosphatidic acid ligand. A total of 148 conotoxin structures were characterized for their binding abilities against LPAR6. Subsequently, top 10 conotoxins were selected on the basis of binding energy values, residual contributions and conformational cluster saturations. BuIA (a member of Alpha- conotoxins family), contryphan-R and contryphan-Lo (Synthetic class) conotoxins, exhibiting efficient binding parameters were subjected to molecular dynamics simulation assays and topology analysis. We propose that BuIA might be a potent antagonist due to its predominant binding at the extracellular region of LPAR6. Current study provides a backbone for understanding structural and functional insights of LPAR6 and findings of this study may be helpful in designing novel therapeutic targets for the treatment of cancers caused by elevated LPAR6 expression.

## 1. Introduction

Lysophosphatidic acid (LPA) is an extracellular and naturally occurring phospholipid mediator that interacts with G-protein coupled transmembrane receptors (GPCRs) and activates multiple cellular processes such as apoptosis, morphogenesis, differentiation, motility and cell proliferation. LPA receptor subtypes (LPAR1, LPAR2 and LPAR3) of endothelial differentiation gene (*Edg*) family are structurally distinct from the members of non-Edg receptor family (LPAR5 and LPAR6). The atypical purine receptor, LPAR6 (P2RY5) is recently identified purinergic receptor that is ubiquitously expressed at a relatively low level in multiple organs except placenta, head and neck [1]. LPAR6 activation via G-protein coupling activates diverse signaling cascades to induce a wide range of fundamental biological functions including forebrain development in *Xenopus laevis*, cytoskeletal changes and hair development in mammals [2–4]. Involvement of LPA signaling in various cellular responses demonstrates that its altered expression may be implicated in several disease states [5].

Recently, it has been reported that LPAR6 sustains proliferation capacity and tumour growth by transcriptional activation of a proto-oncogene *Pim-3* in the liver cancer patients [6]. Similarly, depletion of Lysine-specific demethylase 1 (LSD1) and elevated expression of matrix metallopeptidase-9 (MMP-9) leads to an enhanced expression of LPAR6 in hepatocellular carcinoma [7, 8]. Higher level of LPAR6 correlates with increased migration, invasion and tumour reoccurrence in the androgen independent prostate cancer cells [7]. LPAR6 is upregulated in Acute Myeloid Leukemia (AML) with the t(8;21) translocation resulting in squamous cell carcinomas of skin, testis and bladder [1, 9]. Thus LPAR6 may serve as a promising therapeutic target for the treatment of various cancer types.

Recent potential therapeutic approaches aimed at antagonizing LPARs have gained considerable attention. In this regard, multiple small-molecules (lipid-like; similar to natural ligands and non-lipid) reported for their antagonistic activities against LPARs have entered into clinical trials. Among these, Ki16425/Ki16198 or Debio-0719 blocks LPA induced tumour metastasis through varied mechanism in the hepatocellular carcinoma patients [10–13]. Another LPA_1/3_ antagonist VPC12249 has proven efficacious in the idiopathic pulmonary fibrosis (IPF) studies [14, 15]. BMS-986202, an LPA_1_ inhibitor has successfully completed phase-1 trials for fibrosis. However, to the best of our knowledge, there is still a lack of potent and selective lipid or non-lipid modulators for LPAR6 [16].

The renewed focus of pharmaceutical industry on the drugs isolated from biological sources has coincided with the exploration of animal venom; an unexploited natural resource of small and pharmacologically active peptides. This large source provides novel leads for the development of new therapeutics. Thus molecular specificity and high affinity of these bioactive peptides make them invaluable research tools for pharmacological studies. The best example of peptide toxin biodiversity is the recently evolved conotoxins originated from the venomous marine snails of the genus *Conus*. There are approximately 700 species of cone snails with each producing 1000 conotoxins. Their structural and pharmacological properties including small size, target selectivity and ease of synthesis have contributed to their broadly evolved bioactivity and well-renowned therapeutic potential [17]. Conotoxins constitute extensive combinatorial library of disulphide bridged short peptides, which are capable of simultaneously targeting diverse range of ion channels and other receptor proteins such as G-protein coupled receptors (GPCRs). Pharmacological characterization of T-family conotoxins (LiC32 and τ-CnVA) indicates that they selectively antagonize somatostatin-3 receptor subtype [18]. αC-conotoxin, PrXA and A-superfamily conotoxins including Vc1.1, RgIA, PeIA and AuIB have been reported to competitively antagonize nicotinic acetylcholine receptors [18, 19]. A-superfamily conotoxin (ρ-TIA) has been shown to inhibit α1-adrenoceptor, a GPCR [20]. In another study, eight non-disulphide bridged conotoxins (5 conopressins and 3 contulakins) interact with vasopressin, neurotensin, melanocortin or somatostatin GPCRs [21–23].

Based on these observations, we applied structure-based virtual screening and molecular dynamics simulation assays to evaluate the therapeutic potential of conotoxins against LPAR6. Overall, targeting LPAR6 will offer an opportunity for enhancing drug efficacy in cancer, especially in squamous cell carcinoma and metastatic prostate cancer.

## 2. Material and methods

### 2.1. Data set

The crystal structure of human LPAR6 was obtained through homology modelling as described earlier [24]. The binding pocket details of LPAR6 were characterized by PDBsum [25]. Experimentally known NMR structures of 148 conotoxins (S1 Table) were retrieved through PDB (http://www.rcsb.org/pdb) to characterize their binding abilities with LPAR6. Conotoxins are categorized in various pharmacological classes (alpha, kappa, chi, rho, mu, omega, delta, epsilon, iota, synthetic and unclassified) and superfamilies depending on their types of interactions with targets and similarity profile of their endoplasmic reticulum (ER) signal sequence. The details of their existence, properties and families were accessed via ConoServer [26]. The sequence-structure relationships of conotoxins were monitored through UCSF Chimera [27].

### 2.2. Evolutionary history

In order to ascertain the conserved segments in conotoxins and their evolutionary history, multiple sequence alignment (MSA) analysis was performed by MEGA [28] with 1000 bootstrap replications. Subsequently, neighbour-joining tree was generated by P-distance method. Inkscape 0.91 (https://inkscape.org/en/) graphic editor was utilized to visualize and edit the resulting alignment.

### 2.3. Molecular docking analysis

3D structure of LPAR6 was subjected to docking analysis against 148 conotoxins through AutoDock 4.2 [29] on a OpenSUSE 11.2 containing Intel(R) Core (TM) i5-2300 CPU system (S1 and S2 Tables). Briefly, Polar hydrogen atoms were added and Kollman charges were assigned to ligands and number of torsions was set to zero in order to perform docking experiments with a rigid receptor and flexible ligand. The grid size was set at 50×48×76 Å in the *x-*, *y-*, and *z*-axis, respectively, with 1Å grid spacing to cover the whole receptor. The number of runs for each docking experiment was set to 100. Empirical free energy function and Lamarckian genetic algorithm (LGA) were applied with the following parameters: a population of 100 randomly placed individuals, a maximum number of 27,000 generations, a mutation rate of 0.02, a crossover rate of 0.80, while the remaining docking parameters were set to default. The program automatically grouped potential receptor-ligand complex conformations into clusters based on their RMSD (root mean square deviation) profiles, using the default threshold (2.0 Å).

The docking results were visualized and analysed using UCSF Chimera. Top 10 docked complexes for LPAR6 and conotoxins were selected on the basis of least free energy binding values and cluster saturation. The hydrogen bonding pattern and hydrophobic interactions in these complexes were studied by LigPlot [30]. The residual contributions of LPAR6 and conotoxins were monitored by Accelrys Discovery Studio Visualizer 4.2 [31].

### 2.4. Molecular dynamics simulation assay

To assess the time-dependent behaviour and conformational readjustments in LPAR6 upon binding to conotoxins of diverse background, Molecular dynamics (MD) simulation assays were performed using the three complexes (LPAR6-BuIA, LPAR6-contryphan-R and LPAR6-contryphan-Lo). Groningen Machine for Chemicals Simulations (GROMACS) 5.0.7 package was used to perform MD simulation assays [32]. All simulations were carried out through Dell Precision workstation T7600 system containing Linux Ubuntu 14.04 operating system. The binding patterns were carefully analysed using LigPlot and Accelrys Discovery Studio Visualizer 4.2 [31].

MD simulations were performed using Amber force field [33] to acquire the equilibrated system. Systems were solvated using Tip4p water model [34] in a periodic box using a minimum distance of 1.4 nm, followed by energy minimization (steepest descent algorithm for 500 steps) via a tolerance of 1000 kJ/mol Å^2^ to remove initial steric clashes. Subsequently, appropriate number of counter ions was added to neutralize the system. The energy-minimized systems were treated for 1000 ps equilibration run under constant pressure and temperature conditions. Finally, MD simulations were run for 40 ns time scale under constant temperature (300 K) and pressure (1 atm) using the Berendsen thermostat and barostat. Fast smooth Particle-Mesh Ewald (PME) summation [35] was used to analyse long-range electrostatic interactions with a cut off of 1 nm for the direct interaction. PDB files were generated for every 3 ns interval to monitor the conformational changes. Stability and time-dependent behaviour of each system was investigated at various time scales. GROMACS modules were utilized to analyse the structure stability, residual fluctuations, interactions and compactness of bound systems. DSSP tool embedded in GROMACS package was utilized to analyse secondary structure variations at each time frame.

### 2.5. Membrane topology analysis

In order to study positioning of LPAR6 protein in the lipid bilayer upon binding with conotoxins relative to protein structure using TMDET [36] tool. The coordinates of LPAR6-conotoxin complexes at different time intervals of MD simulations were utilized for membrane localization studies through Protter [37].

## 3. Results

### 3.1. LPAR6 structural detail

The evaluation tools showed the efficacy and reliability of LPAR6 structure. ProCheck demonstrated 95.5%, 3.8% and 0.3% of non-glycine and non-proline residues in the most favoured, additionally allowed and generously allowed regions of the Ramachandran plot. Occurrence of >98% residues in the favourable region of Ramachandran plot implies the reliability of LPAR6 structure. Three binding cavities predicted by PDBsum were shown at the surface of LPAR6 in S1 Fig. Volume of largest binding cavity was roughly around three times the size of remaining two cavities.

In this study, 148 structurally identified conotoxins (50 alpha, 8 chi, 5 delta, 1 epsilon, 2 iota, 3 kappa, 11 mu, 18 omega, 17 chemically modified and 29 unclassified) were evaluated at sequence and structure levels. Multiple sequence alignment (MSA) of these conotoxins was carried out to determine their evolutionary relationships and extent of sequence conservation (S2 Fig). A higher bootstrap replication value in neighbour joining tree ensured the reliability of topology. Majority of conotoxins having similar pharmacological properties were clustered together in the phylogenetic tree (S2 Fig). However, chemically modified and unclassified conotoxins exhibited diverse clustering patterns.

### 3.2. Docking analysis

**Fig 1:**
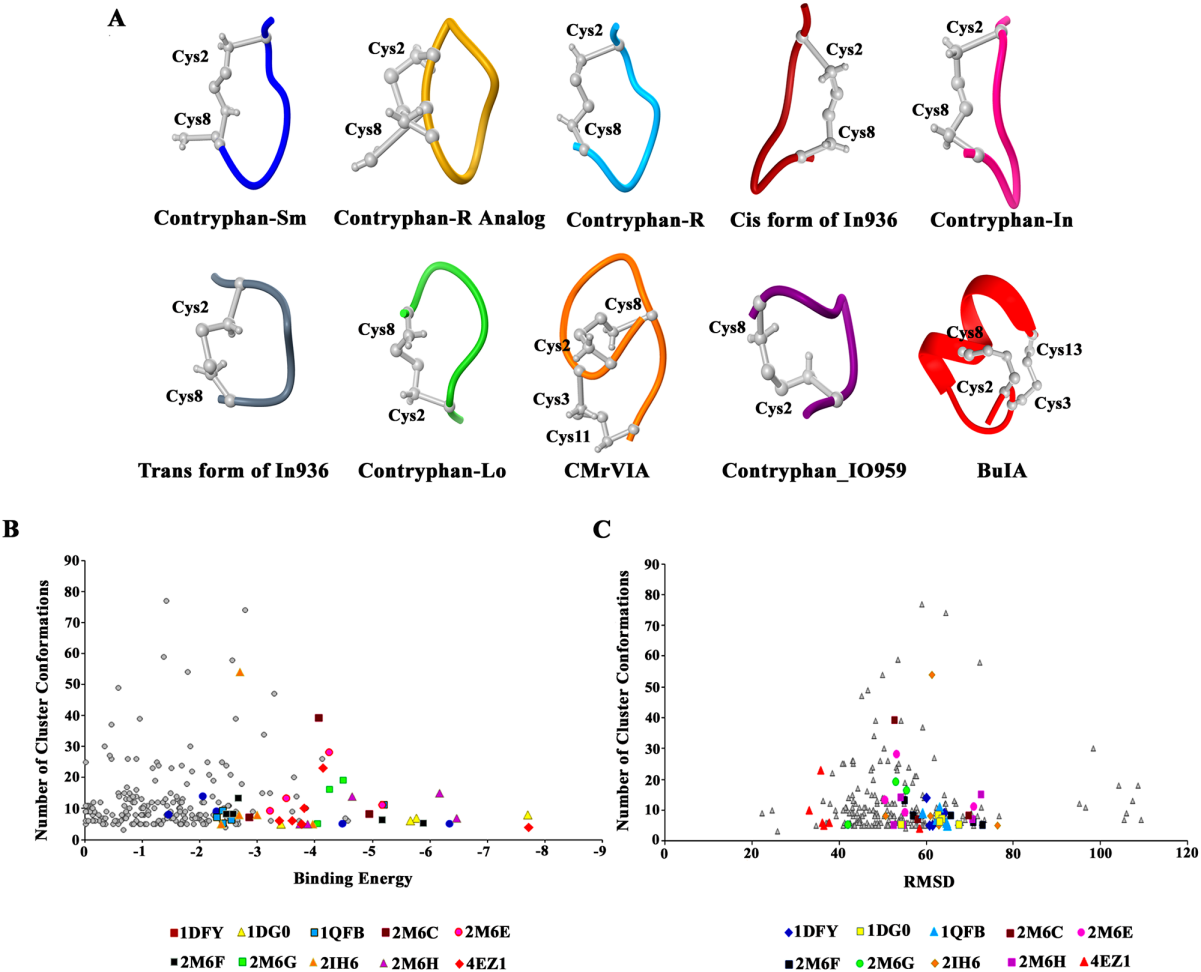
**(A) Three dimensional structures of selected conotoxins. (B) Plot of binding energies versus number of cluster conformations**. Each point in the graph represents total conformations in the cluster and binding energy value of the least energy conformation in that cluster. Conformations of top 10 least scoring energy complexes are highlighted in various colours. **(C) Plot of cluster RMSDs versus number of cluster conformations**. Each data point represents the total number of cluster conformations and root mean square deviation among them. Conformations of top 10 least scoring energy complexes are indicated in different colours.

3D structures (PDB IDs listed in S1 Table) of conotoxins were utilized for molecular docking analysis against LPAR6 to monitor their binding capabilities. The details of binding energy values and cluster RMSDs of docking poses are listed in S2 Table. The distribution of binding energies and RMSDs corresponding to all energetically favourable conformations for conotoxins are graphically represented in Fig 1.

**Fig 2:**
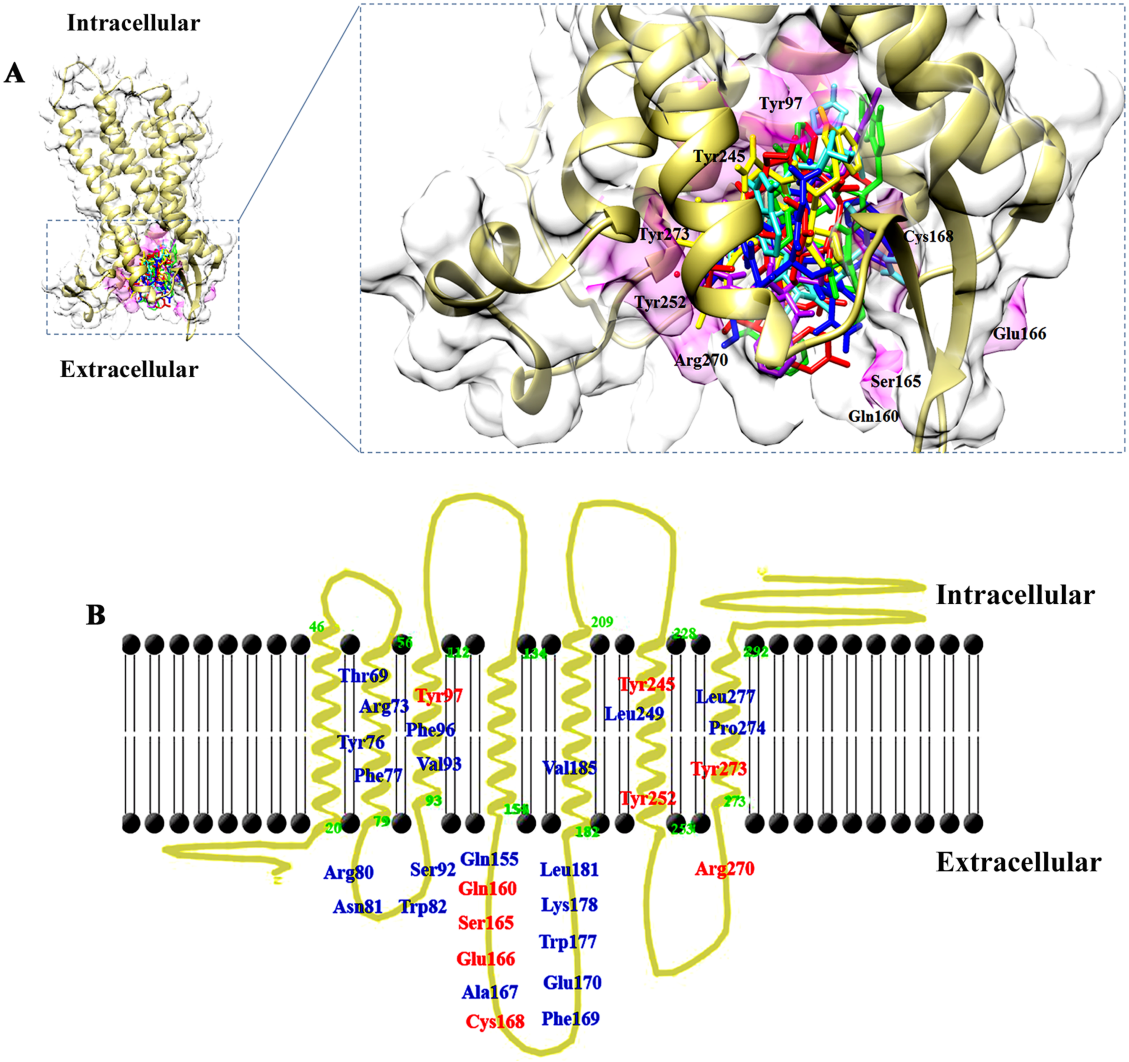
**(A) LPAR6-binding pocket shared by 10 selected conotoxins**. Binding pocket is indicated in magenta, while interacting residues are represented in black colour. The bound conotoxins are depicted in stick representations. (**B) LPAR6 specific residual contributions in conotoxins binding**. LPAR6 embedded in the plasma membrane is shown in goldenrod, while residues involved in hydrogen bonding and hydrophobic associations are indicated in red and blue colours, respectively.

10 conotoxins (BuIA (4EZ1), Des [Gly1] contryphan-R (1DG0), contryphan-R (1QFB), contryphan-Lo (2M6G), contryphan_IO959 (2M6H), contryphan-Sm (1DFY), Cis form of In936 (2M6C), Trans form of In936 (2M6F), contryphan-In (2M6E) and CMrVIA (2IH6) were selected on the basis of least binding energy values and saturated clusters (Fig 2). Three most probable binding regions of LPAR6 in contact to conotoxins were Tyr76-Trp82, Gln160-Phe169 and Arg270-Tyr273. It was observed that Arg73, Arg76, Phe77, Arg80, Trp82, Cys168, Phe169, Leu181, Tyr252, Arg270 and Tyr273 in LPAR6 exhibited stable interactions with 6, 7, 8, 8, 6, 9, 9, 6, 6, 7 and 10 docked conotoxins (Table 1). In addition to these residues, Val93, Gln160, and Trp177 residues of LPAR6 were also detected in binding.

**Table 1:** PDBs of ten top scoring conotoxins forming hydrophobic contacts and hydrogen bonding with LPAR6 residues. Peptide IDs in bold are implicated in hydrogen bonding with LPAR6.

PDB IDs of conotoxins	LPAR6 binding residues
2M6H, 2M6G	Thr69
2M6H, 2M6G, 2M6F, 2M6E, 1QFB, 1DG0	Arg73
4EZ1, 2M6F, 2M6E, 2M6C, 1QFB, 1DG0, 1DFY	Tyr76
2M6H, 4EZ1, 2M6G, 2M6F, 2M6E, 1QFB, 1DG0, 1DFY	Phe77
4EZ1, 2M6G, 2M6F, 2M6C, 2IH6, 1QFB, 1DG0, 1DFY	Arg80
2M6C, 1QFB, 1DFY	Asn81
4EZ1, 2M6G, 2M6F, 2M6C, 1QFB, 1DFY	Trp82
2M6H, 2M6G	Ser92
2M6H, 2M6G, 2M6F, 2M6C, 1QFB	Val93
2M6H, 4EZ1, 1QFB, 1DG0	Phe96
2M6E, 2M6C, 1DG0, **4EZ1**	Tyr97
1QFB	Gln155
4EZ1, 2M6F, 2M6C, 1DFY, **2IH6**	Gln160
**4EZ1**	Ser165
2M6G, 1DFY, **1QFB**	Glu166
2M6G, 2IH6, 1QFB	Ala167
4EZ1, 2M6G, 2M6E, 2M6C, 2IH6, 1DG0, 1DFY, **2M6F, 1QFB**	Cys168
4EZ1, 2M6G, 2M6F, 2M6E, 2M6C, 2IH6, 1QFB, 1DG0, 1DFY	Phe169
2M6E, 1DG0, 1DFY	Glu170
2M6E, 2M6C, 2IH6, 1QFB, 1DFY	Trp177
2M6C, 2IH6	Lys178
4EZ1, 2M6C, 2IH6, 1QFB, 1DG0, 1DFY	Leu181
1DG0	Val185
1QFB, 1DG0, **2M6G**	Tyr245
4EZ1, 1QFB, 1DG0	Leu249
2M6H, 4EZ1, 2M6C, 2IH6, **1DG0**, 1DFY	Tyr252
2M6H, **4EZ1**, 2M6G, 2M6F, 1QFB, 1DG0, 1DFY	Arg270
2M6H, 4EZ1, 2M6G, 2M6F, 2M6E, 2M6C, 2IH6, **1QFB**, 1DG0, 1DFY	Tyr273
2M6H, 4EZ1, 2M6E, 1QFB	Pro274
2M6H, 4EZ1, 2M6E, 1DG0	Leu277

The docking analysis revealed that all conotoxins bind at the extracellular region of LPAR6 (Fig 2). Nevertheless, it is unclear at the moment whether binding of peptides results in any noteworthy alteration in the function of LPAR6. The extracellular residues (Gln160, Ser165, Glu166, Cys168 and Arg270) were involved in hydrogen bonding with conotoxins, while residues involved in hydrophobic associations were located in the transmembrane and extracellular regions of LPAR6 (Fig 2).

The selected peptides were analysed for their drug-like properties through ProtParam tool [38]. Biological properties such as molecular weight, isolectric point, instability index, extinction coefficient and estimated half-life of these conotoxins are shown in S3 Table. Instability index values < 40 indicated that all peptides except 1DG0, 1QFB and 1DFY were quite stable (S3 Table). Values derived from grand average of hydropathy (GRAVY) demonstrated that 4EZ1 and 2M6E peptides might be able to cross the hydrophobic transmembrane barrier. Estimated half-life of all the peptides was similar (30 hours) in yeast and *E*.*coli*. Extinction coefficient explains the light absorbance ability by a protein. Approximately, similar values of extinction coefficient for 4EZ1 and 2IH6 highlighted the ease of purification process associated with their synthesis.

### 3.3. Molecular dynamics simulation analysis

Apo and conotoxin-bound LPAR6 complexes (LPAR6-BuIA, LPAR6- contryphan-R and LPAR6- contryphan-Lo) were subjected to molecular dynamics (MD) simulations to elucidate the dynamic behaviour of LPAR6 and to gauge the pattern of system stability upon binding to conotoxins. Dynamic trajectories of each simulated system were thoroughly investigated to assess the stability and conformational changes by plotting the RMSD (Root mean square deviations), RMSF (Root mean square fluctuations), Rg (Radius of gyration), RDF (Radial distribution function), hydrogen bonding and secondary structure plots.

**Fig 3:**
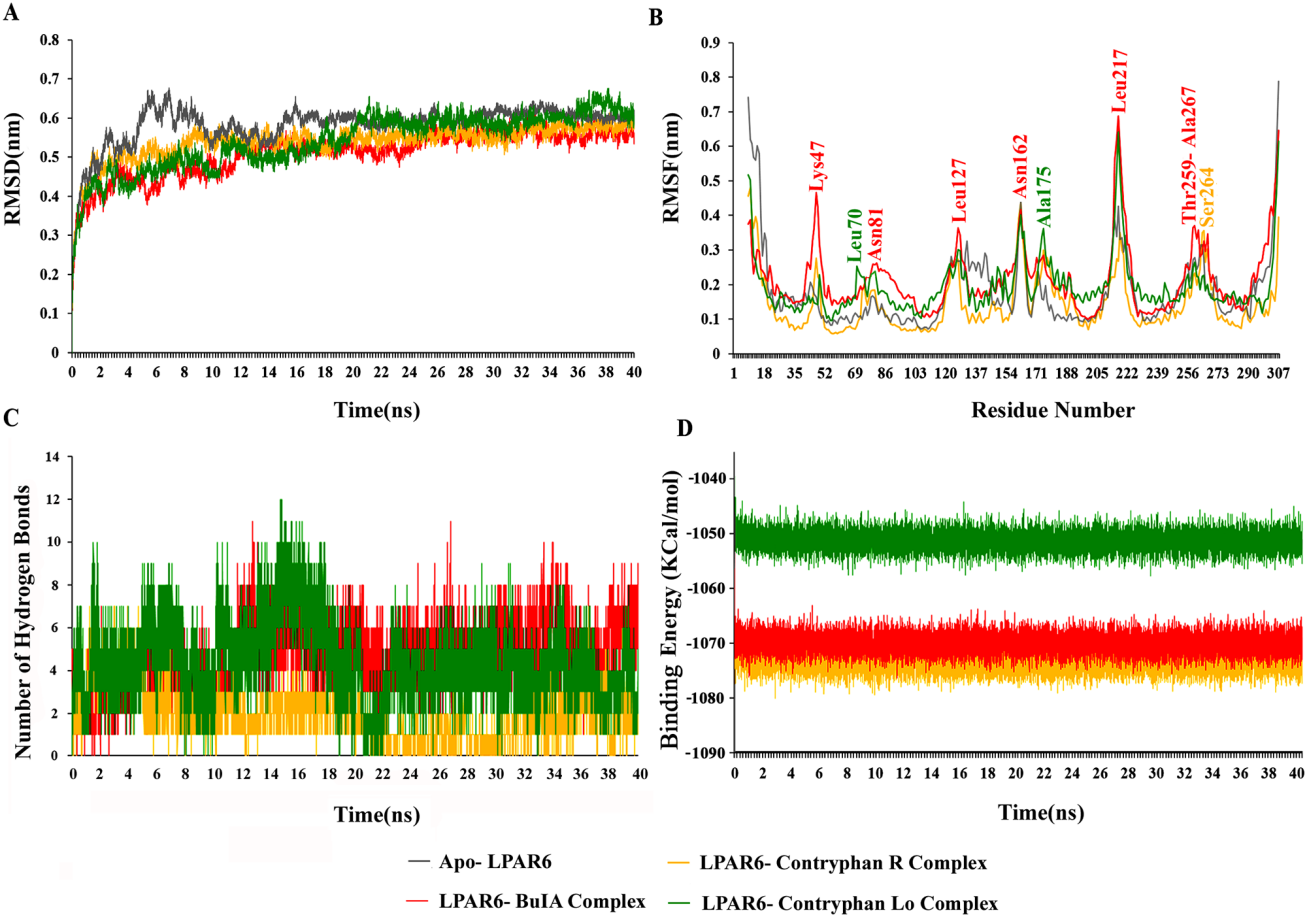
RMSD, RMSF, inter molecular hydrogen bonds and binding energy plots for 40 ns MD simulations to investigate stability and fluctuation of bound and apo-LPAR6. Apo-LPAR6 and its bound forms (apo-LPAR6, LPAR6-BuIA, LPAR6- contryphan-R and LPAR6- contryphan-Lo) are illustrated in grey, red, golden rod, and green colours, respectively. (A) RMSD plots were computed through least square fitting of backbone Cα-atoms. (B) Comparative RMSF plots of apo-LPAR6 (grey), LPAR6-BuIA (red), LPAR6- contryphan-R (goldenrod) and LPAR6- contryphan-Lo (green). More fluctuating residues are labelled in the corresponding colours. (C) Intermolecular hydrogen bonds of LPAR6 specific residues in complex with BuIA, contryphan-R and contryphan-Lo. (D) LJ-SR binding energy plot for 40 ns MD simulation.

The overall stability of each complex was measured by estimating the RMSD profile that showed a quite stable interaction behaviour at 10 ns time scale. On average, RMSD values of system derived C-alpha atoms varied between 5-6Å for all systems (Fig 3A). Rg analysis revealed a variation in the compactness of systems. Generally, a higher Rg value implies lower compactness of a system. Highly stable and least varying Rg trend was prominent in the LPAR6-BuIA complex, while in contryphan-R and contryphan-Lo complexes, relatively lower Rg values were observed as represented in S3 Fig. Consequently, LPAR6 bound to BuIA exhibited less tight packing as compared to apo-form. In contrast, contryphan-R exhibited more compactness. LPAR6- contryphan-Lo complex exhibited more variation in the compactness during 20–30 ns time scale. These data indicated that structural transitions in BuIA resulted in less tight packing, while in case of contryphan-R and contryphan-Lo, LPAR6 binding induced more compaction in the structure. Accordingly, higher Rg values of bound complexes (except within 19–28 ns) than that of apo-LPAR6 suggested the firmness in LPAR6 associated with BuIA, contryphan-R and contryphan-Lo, due to the conformational adaptations.

RMSF plots indicated the presence of residual flexibility upon LPAR6 binding to conotoxins (Fig 3B). All the loop specific residues (Val45-Asn50, Arg80-Phe84, Tyr120-Thr126, Gln160-Ser165, Lys213-Lys221, and Val266-Val269) in LPAR6 showed a higher fluctuation rate throughout the simulation time. In case of LPAR6-BuIA and LPAR6-contryphan-Lo, Leu217 showed more fluctuations (6.8Å and 6.4Å, respectively) as compared to apo form. LPAR6 critical residues involved in contryphan-R, BuIA and contryphan-Lo binding (Arg73, Arg80, Gln160, Ser165, Cys168, Glu170, Tyr252, Arg270 and Tyr273) exhibited lower RMSF values ranging between 1.1Å- 3Å.

The binding characteristics of LPAR6 with BuIA, contryphan-R and contryphan-Lo were examined through plotting time-dependent intermolecular hydrogen bonds. Overall, LPAR6-BuIA and LPAR6-contryphan-Lo complexes exhibited more hydrogen bonds (Fig 3C), indicating a greater strength of associations with LPAR6. Binding free energy (LJ-SR) calculation indicated that LPAR6 bound conotoxins exhibited stable binding energy values (-1033 to -1080 kcal/mol). LPAR6-contryphan-R complex exhibited lowest energy values, followed by LPAR6-BuIA complex (Fig 3D).

**Fig 4:**
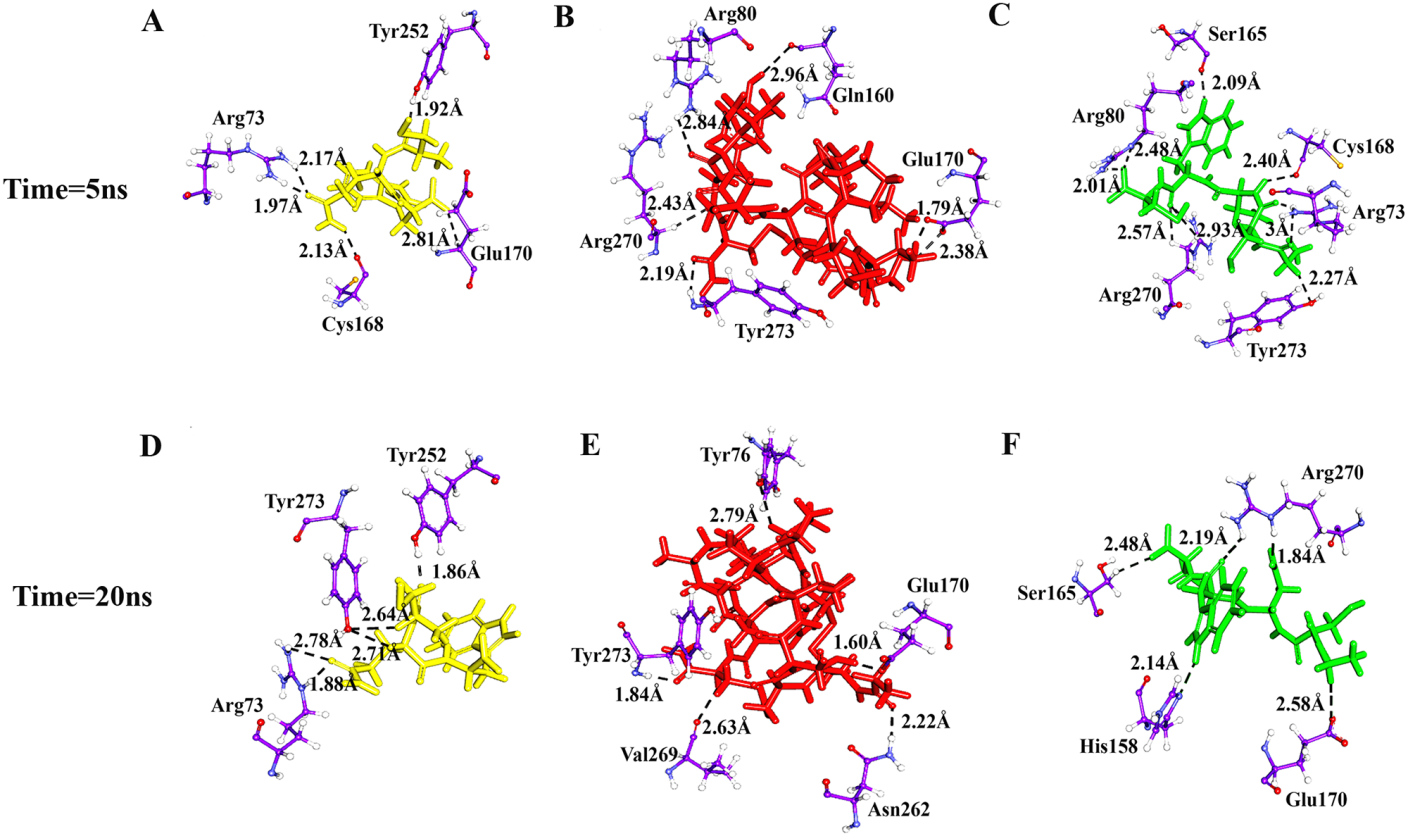
Binding mode and molecular interaction analyses of conotoxin with LPAR6. Simulated complexes of (A and D) LPAR6- contryphan-R, (B and E) LPAR6-BuIA and (C and F) LPAR6- contryphan-Lo at 5 ns and 20 ns time scales, respectively. Interacting residues of LPAR6 are shown in purple ball and sticks, while BuIA, contryphan-R and contryphan-Lo are shown in stick mode in red, goldenrod and green colours, respectively. Hydrogen bonds are indicated by black dotted lines with distances in angstrom.

Conotoxin-bound LPAR6 complexes achieved stability at 10 ns time scale as evident in RMSD plot (Fig 3). In order to comparatively visualize the residual interactions in LPAR6 during simulation time, PDB files at different time scales were generated. LPAR6 specific Arg73, Tyr76, Arg80, His158, Gln160, Ser165, Cys168, Glu170, Tyr252, Asn262, Val269, Arg270 and Tyr273 residues were involved in hydrogen bonding with conotoxins. Arg73 and Tyr252 residues were found to be consistently interacting with contryphan-R, while Glu170 and Tyr273 residues were associated with BuIA throughout the simulation period. Likewise, Ser165 and Arg270 residues constituted hydrogen bonding with contryphan-Lo. Evidently, no interaction was detected with the transmembrane or intracellular segments of LPAR6. Hence, a clear unambiguous role of LPAR6 extracellular domain was witnessed in the affiliation with conotoxins (Fig 4).

### 3.4. Secondary structure profile analysis

**Fig 5:**
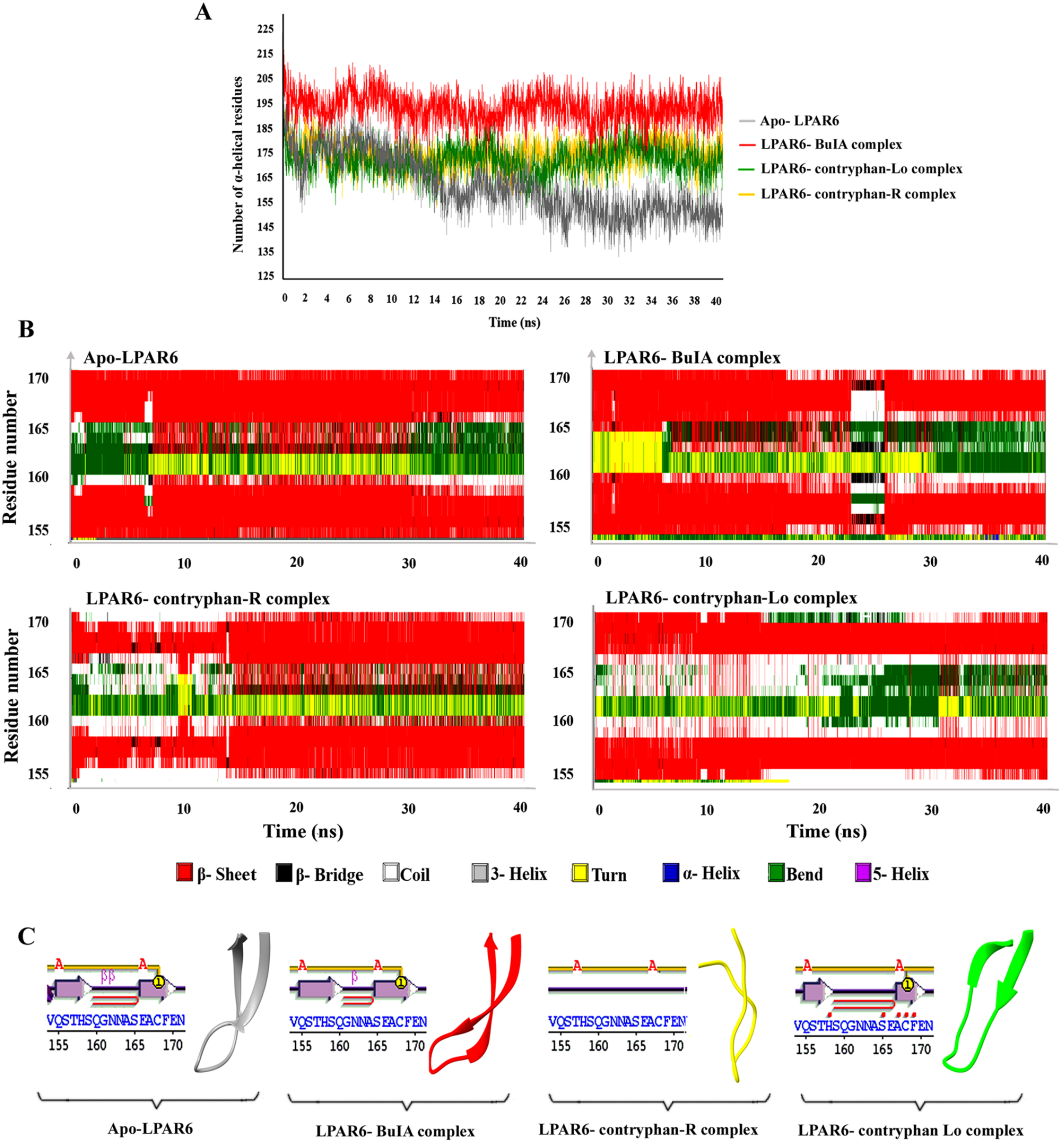
DSSP analysis of simulation trajectories. (A) Time-dependent analysis of MD trajectories to infer the number of alpha helical residues during simulation time period. Apo and bound forms of LPAR6 with BuIA, contryphan-R and contryphan-Lo are represented in grey, red, golden rod and green colours, respectively. (B) Conformational readjustments in the β-sheet region spanning 155–170 residues during MD simulation runs. Time-dependent plots for of apo-LPAR6 and LPAR6-conotoxin complexes. (C) β- sheet regions in apo-LPAR6 and BuIA, contryphan-Lo and contryphan-R bound states of LPAR6 at 5 ns time scale are shown in grey, red, goldenrod and green ribbons for individual complexes.

The DSSP (Define Secondary Structure of Proteins) analysis for three complexes (LPAR6-BuIA, LPAR6- contryphan-R and LPAR6- contryphan-Lo) and apo-LPAR6 was performed to assign time-dependent secondary structures to the LPAR6 residues. A majority of conformational changes were witnessed in the β-sheet specific region (Gln155-Glu170 AA). For example, β-sheet remained preserved in the LPAR6-BuIA complex except during the time scale of 23–26 ns where it dramatically disappeared. However, a clear β-sheet prolongation till 7 ns reflects strong affinity between BuIA and LPAR6 in this critical period (Fig 5B). β-sheet length reduction and an elongation of coil region in LPAR6-contryphan-R complex continued till 14 ns (Fig 5B). In case of LPAR6-contryphan-Lo, a distinct secondary structure amendment was visualized where clear extension in the intertwining coiled region encompassing Gln159-Glu166 residues was maintained throughout the MD simulation trajectory (Fig 5B). A snapshot of bound and unbound states of LPAR6 in the simulated systems frozen at 5 ns represents evident differences in the length and orientation of β-sheet (Fig 5C). These observations supported that large structural alterations in β-sheet region of bound complexes accommodated the conotoxins binding.

LPAR6 residues implicated in α-helical regions were analysed using DSSP tool to investigate the residual contributions in the simulation time period (Fig 5A). Evidently, a sharp contrast among bound and unbound systems was observed with reference to the α-helical conformation. Apo-LPAR6 exhibited a drastic decrease in the α-helical length at 10 ns, compared to the bound form of LPAR6. A significant difference of 60 α-helix shaping residues during starting and ending phases of simulation reflect highly unstable behaviour of unbound LPAR6 over the period of time (Fig 5A). Correspondingly, conotoxin-bound LPAR6 depicted slight variations in the α-helical content over the simulation time period.

### 3.5. Membrane topology studies

**Fig 6:**
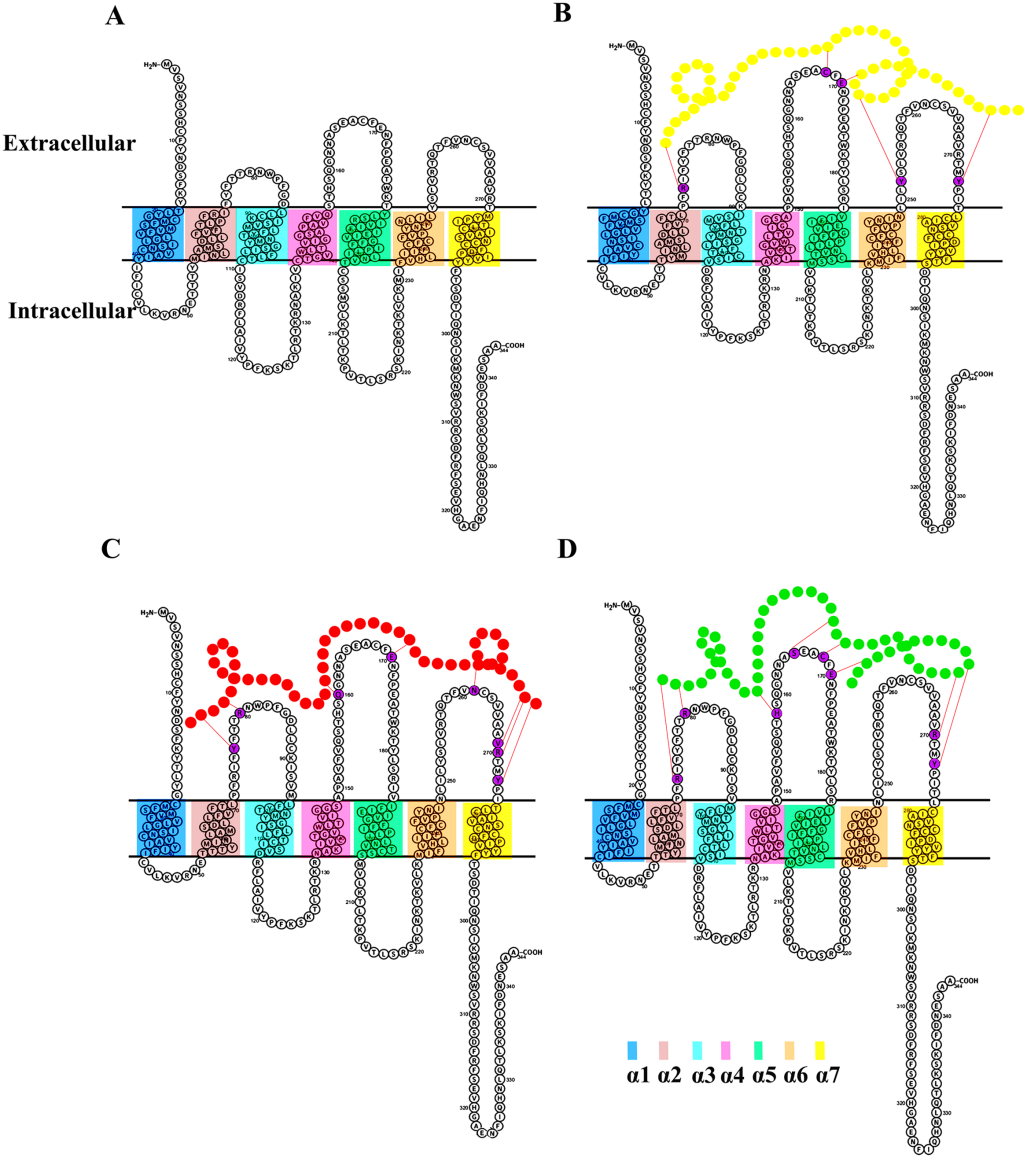
Topology changes in the transmembrane helices of LPAR6 after interacting with conotoxins. Membrane localization of α-helical regions for (A) Apo LPAR6, (B) LPAR6-contryphan-R (C) LPAR6-BuIA, and (D) LPAR6- contryphan-Lo complexes. BuIA, contryphan-R and contryphan-Lo peptides are indicated in red, yellow and green colours respectively. Hydrogen bonds are mentioned by red coloured dotted lines and the interacting residues of LPAR6 are shown in purple colour.

Based on the observation that alterations in the α-helical lengths result in the variable topology of transmembrane regions within the hydrophobic layer of plasma membrane, comparative topological changes in the seven transmembrane segments of LPAR6 were monitored in bound and unbound systems (Fig 6). These changes were predicted at 20 ns by analysing the coordinate files in TMDET. LPAR6 N-terminal region was elongated in conotoxin-bound complexes. Similarly, compression of LPAR6 C-terminal region was prominent due to binding with conotoxins as compared to apo form. Significant changes in the sizes of LPAR6 extracellular segments were evident as these particular regions were implicated in the conotoxin interaction. In contrast, intracellular segments remained unaltered except in case of LPAR6-BuIA complex where a clear shortening of second intracellular segment (Arg141-Arg131 in comparison to Ile110-Val137 in apo-LPAR6) was witnessed (Table 2). Extracellular regions in the bound complexes exhibited almost similar lengths. β-sheet extended to great degree in all bound forms of LPAR6 as compared to the apo state. For example, β-sheet resized from 156-179AA in apo-LPAR6 to 149-185AA in LPAR6-BuIA at 20ns interval (Fig 6).

**Table 2:** Conformational alterations in the lengths of LPAR6 extra and intra cellular segments upon conotoxins binding at 20ns interval. IC and EC denote intracellular and extracellular regions, respectively.

	IC1	EC1	IC2	EC2	IC3	EC3
**Apo-LPAR6**	Ile41- Tyr55	Phe75- Asp86	Ile110- Val136	Ser66- Thr179	Cys203- Ile231	Tyr250- Tyr271
**LPAR6-contryphan-R**	Cys44- Tyr53	Pro71- Lys90	Asp113- Asn132	Pro150- Ile184	Val207- Leu228	Leu249- Tyr276
**LPAR6-BuIA**	Cys44- Glu51	Pro70- Met94	Arg114- Arg131	Ala149- Val185	Met206- Lys229	Asn248- Ile275
**LPAR6-contryphan-Lo**	Val45- Tyr52	Pro71-Val93	Asp113- Arg131	Ala149- Arg183	Val207- Leu228	Asn248- Leu277

## 4. Discussion

LPAR6 mediates cell viability by possessing pro-survival activity in the cancer cells. Enhanced LPAR6 signalling promotes migration of prostate cancer cells and its overexpression is associated with squamous cell carcinomas of the lung, cervix, skin, urinary bladder, testis, head and neck [1]. LPAR6 is involved in maintaining proliferation capacity and tumorigenic phenotype in the hepatocellular carcinoma [6]. Thus targeting of LPAR family member offers a promising opportunity to enhance drug efficacy in cancers, particularly in the squamous cell carcinoma [1].

In recent years, strategies aimed at antagonizing LPA receptors have gained considerable attention. The antagonists include lipid-like molecules that share high structural similarity with natural ligand LPA and other non-lipid chemical entities. Despite our growing functional knowledge of LPA receptors, the number of currently available efficient antagonists is still low and none of the LPAR6 targeting drugs has been FDA approved yet [39]. Though several antagonists (dioctylglycerol pyrophosphate, fatty alcohol phosphate, VPC12249 and Ki16425) have been developed for LPA_1-3_ receptors [5], no evidence exists for the discovery of non-lipid antagonists of LPAR6 [16]. In view of these facts, current study explored the binding characteristics of LPAR6 and conotoxins derived from the cone snail venom through *in silico* approaches.

BuIA (a member of Alpha conotoxin family), contryphan-R and contryphan-Lo (synthetic) conotoxins exhibited binding affinities at the extracellular region of LPAR6. The occupancy of bound conotoxins was elaborated through MD simulation assays, which demarcated the active contribution of LPAR6 β-sheet region (Gln155-Glu170AA) in the conotoxin binding (Fig 5B). More pronounced topological changes were evident in the EC2 region; sandwiched between the 4^th^ and 5^th^ transmembrane helices of LPAR6 (Fig 6). Particularly, Cys168 and Tyr273 residues lying in the proximity of LPAR6 5^th^ and 7^th^ transmembrane helices assisted in the formulation of the peripheral binding pocket groove. Evidently, shifting of β-strands into loop conformation was visible in contryphan-R bound LPAR6, while shortening of β-strands was observed in case of LPAR6-contryphan-Lo complex (Fig 5B). The compelling evidence of LPAR6 specific residual involvement (Tyr273, Tyr252 and Arg270 and Arg73) in both inhibitor and substrate binding equates the recognition of natural ligands [24] with the antagonist-binding paradigm.

**Fig 7:**
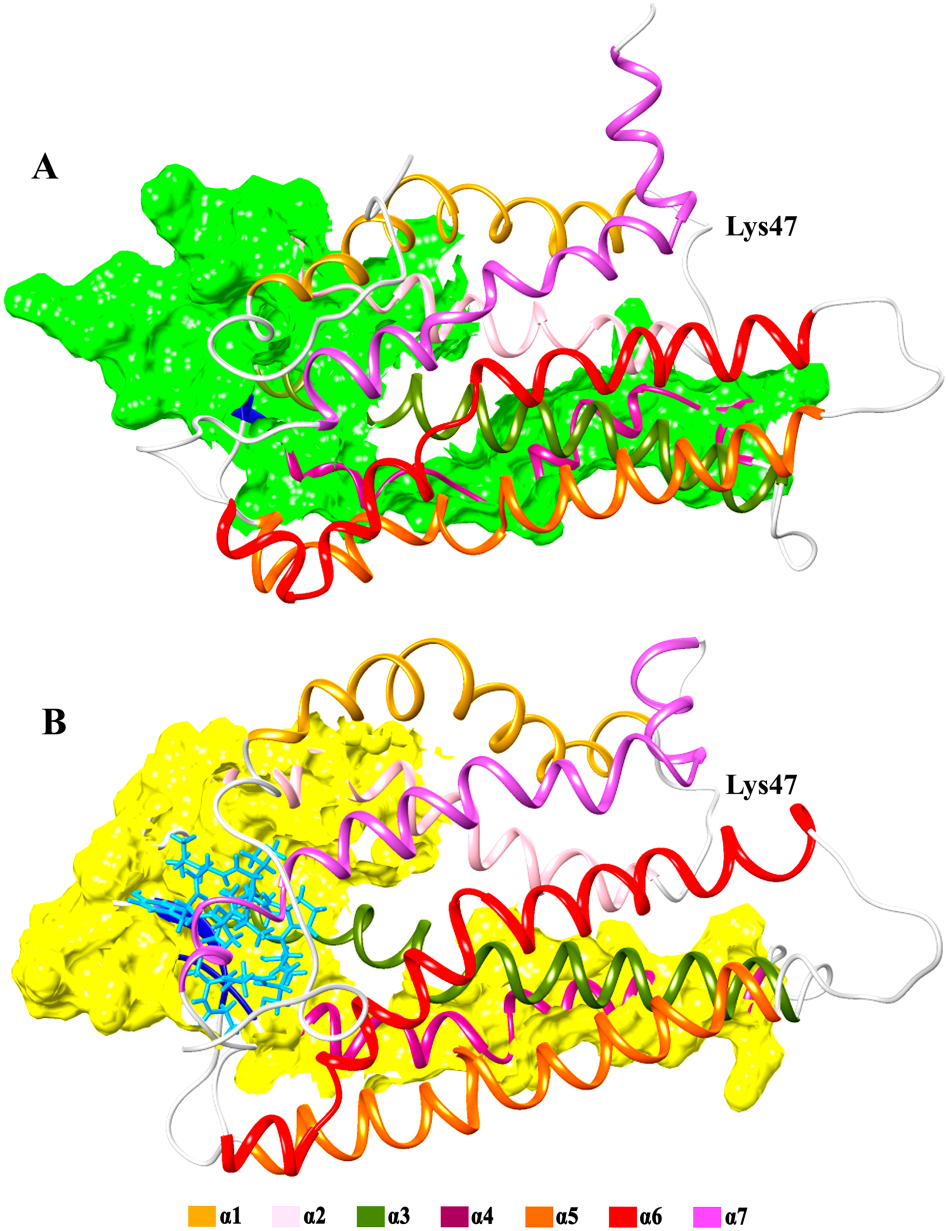
Cavity size difference between A) apo and B) BuIA bound LPAR6. Seven alpha helices in LPAR6 are colored as follows; α1: golden rod, α2: pink, α3: olive drab, α4: violet red, α5: orange, α6: red and α7: orchid. Coils and β-sheets are in light grey and dark blue colors respectively, while bound BuIA atoms are represented in cyan color.

Among the previously reported α-conotoxins bearing antagonistic specificity, BuIA has shown predominant affinity and activity against neuronal nicotinic acetylcholine receptor subtypes [40]. Such properties of BuIA may be attributed due to its unique existence of 4/4-cysteine spacing, a characteristic absent in other peptide members of alpha conotoxins class [41]. It has been reported that globular isomer of BuIA is more stabilized during oxidative refolding due to its disulphide connectivity [42]. Active involvement of Cys13 in constituting hydrogen bond with LPAR6 clearly emphasizes the association of exceptional cysteine bridging in BuIA with its antagonistic affinity against LPAR6 (S2B Fig). Interestingly, Lys47 residue of BuIA-bound LPAR6 exhibited more fluctuation (5Å) as compared to apo-LPAR6, LPAR6-contryphan-R and LPAR6-contryphan-Lo complexes (2Å), resulting in the inward movement of adjacent loop (Fig 3). Consequently, increase in the helical content (Fig 5A) shifted the extracellular regions (EC2 and EC3) to widen the size of LPAR6-specific binding cavity during BuIA binding as evident in Fig 7.

Lowest extinction coefficient (S3 Table), relatively more estimated half-life (S3 Table) and comparatively lower instability index (S3 Table) suggest a stable and permeable nature of BuIA. Moreover, the clues such as similar residual interactions to that of natural ligand for LPAR6 (Table 1 and [24]), preservation of interacting β-sheet throughout the simulation period (Fig 5B), highest number of residues contributing in the α-helical regions (Fig 5A), sustainment of highest number of hydrogen bonds across MD trajectory (Fig 3C), lowest docking score among all conotoxins (S4 Table), and significantly lower binding energy value throughout the simulation timeline (Fig 3D) suggest the potential of BuIA as a strong competitive antagonist of LPAR6. Thus BuIA may serve as a natural peptide-based antagonist to competitively inhibit the LPA binding site of LPAR6 in prostate cancer and squamous cell carcinoma. However, LPAR6 inhibition by BuIA is susceptible to the disruption of lipid rafts, as reported for LPAR1 in another study [42]. Both LPAR6 and LPAR1 share high structure similarity (S4 Fig), it may be plausible that they possess similar activity against lipid rafts.

Further pre-clinical and clinical studies are required to validate the *in-vivo* efficacy of BuIA in prostate cancer lines. The design of nonpeptide mimetic, identification of small molecule mimetics via *in silico* screening of chemical libraries and structure-activity relationships to generate analogs with improved bioavailability will collectively serve as the basis for anticancer drug development.

## Supporting information

S1 Fig **(A) LPAR6 3D structure. (B) Details of LPAR6 specific binding cavities**. The cavity in red colour is located at the extracellular region of LPAR6, while purple and yellow coloured pockets are located in the intracellular and transmembrane region, respectively.
(TIF)

S2 Fig **A) Neighbour-joining tree of 148 conotoxins**. Sequences derived from the PDB structures of all conopeptides are subjected to MSA by MEGA6 to generate a neighbour joining tree. The pie chart in the centre depicts percentage representation of studied pharmacological classes of conopeptides, indicated by respective colours. **B) Multiple sequences alignment of peptides from alpha class of conotoxins**. All peptides of alpha class are aligned in order to have graphical representation of sequence based conservation. Significant residues with similar physiological properties in various positions are represented in specific colors, whereas underlined residues are those forming hydrogen bonding with LPAR6.
(TIF)

S3 Fig Radius of gyration plot to exhibit the compactness of bound and unbound simulated systems. (TIF)

S4 Fig 3D structure alignment of LPAR1 and LPAR6. Modelled structure of LPAR6 is superimposed in 3D space with known structure of LPAR1 (PDB ID: 4Z34). LPAR6 is represented in light blue while LPAR1 is depicted in deep pink. Seven aligned transmembrane segments are numbered from 1 to 7 in black color.
(TIF)

S1 Table Details of explored conotoxins. PDB IDs along with primary sequences and pharmacological classes of considered conotoxins.
(DOCX)

S2 Table Details of docking clusters in conopeptides-LPAR6 minimum scoring energy complexes. Every lowest free energy valued docked complex is observed to record total number of conformation lying in a specific cluster (Clusters) and corresponding energy range of the cluster conformation (Energy Range of Cluster), Average binding energy of the cluster conformations (Binding Energies) and Root mean square deviations between conformations (RMSDs).
(DOCX)

S3 Table Comparison of drug-like properties of selected conopeptides. (DOCX)

S4 Table Comparative binding analysis of LPAR6 bound conopeptides. (DOCX)
