# Supplementary figures and images for: Alpha conotoxin-BuIA globular isomer is a competitive antagonist for oleoyl-L-alpha-lysophosphatidic acid binding to LPAR6; A molecular dynamics study
Source: PLoS One. 2017 Dec 6;12(12):e0189154. doi: 10.1371/journal.pone.0189154 (PMC5718415; doi:10.1371/journal.pone.0189154)

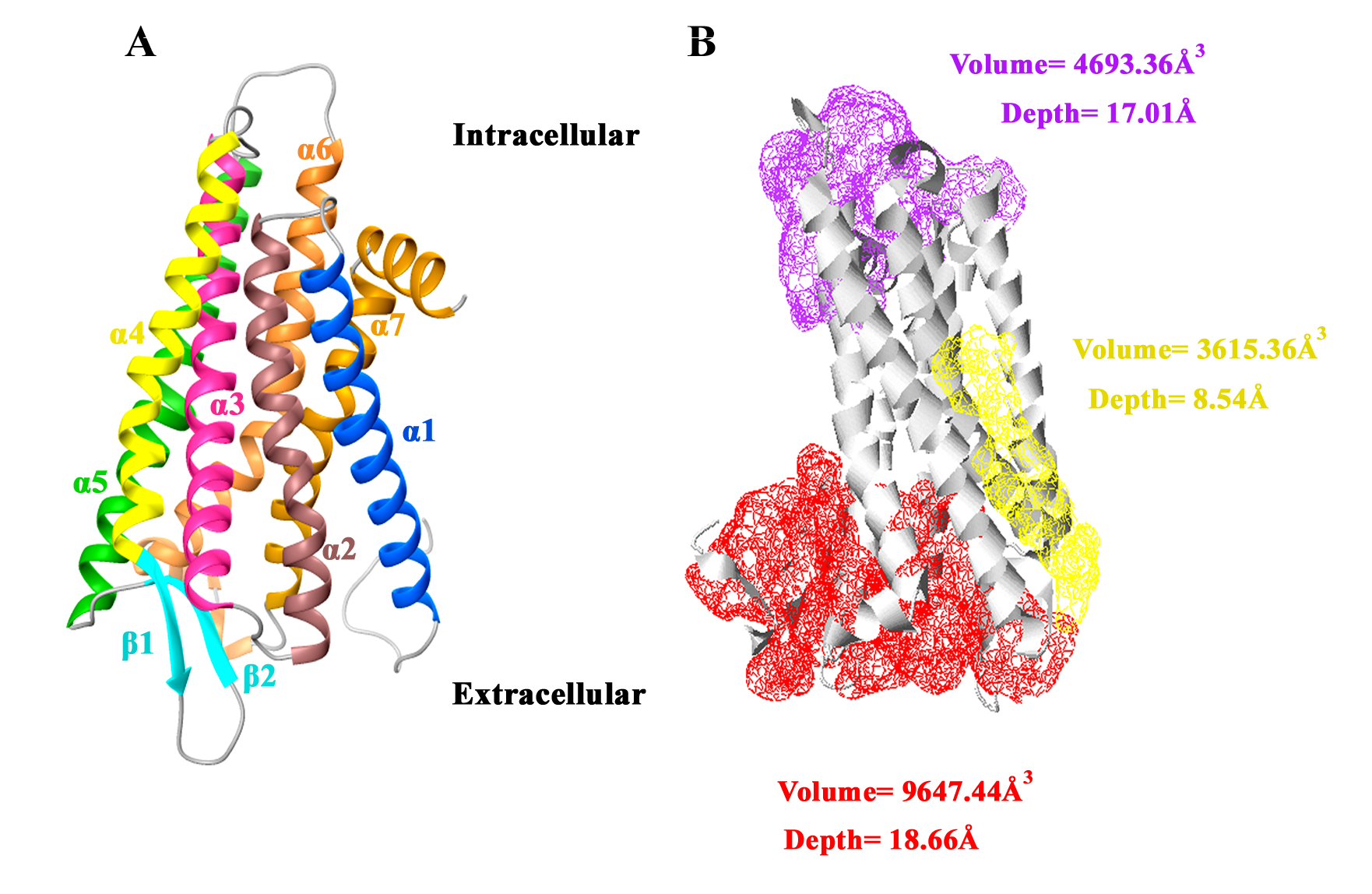

Supplement: S1 Fig — (A) LPAR6 3D structure. (B) Details of LPAR6 specific binding cavities. The cavity in red colour is located at the extracellular region of LPAR6, while purple and yellow coloured pockets are located in the intracellular and transmembrane region, respectively. (TIF) [file pone.0189154.s001.tif]

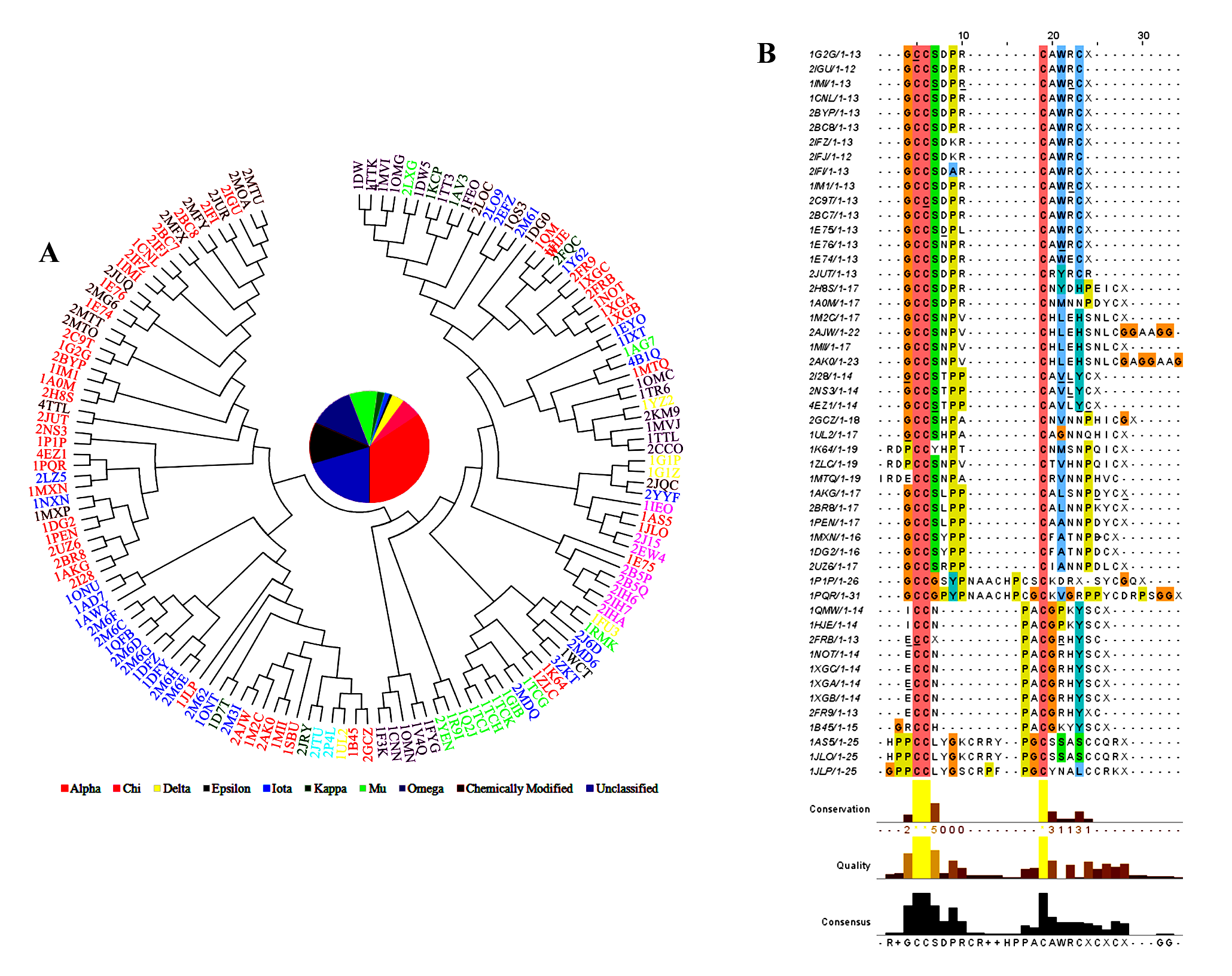

Supplement: S2 Fig — A) Neighbour-joining tree of 148 conotoxins. Sequences derived from the PDB structures of all conopeptides are subjected to MSA by MEGA6 to generate a neighbour joining tree. The pie chart in the centre depicts percentage representation of studied pharmacological classes of conopeptides, indicated by respective colours. B) Multiple sequences alignment of peptides from alpha class of conotoxins. All peptides of alpha class are aligned in order to have graphical representation of sequence based conservation. Significant residues with similar physiological properties in various positions are represented in specific colors, whereas underlined residues are those forming hydrogen bonding with LPAR6. (TIF) [file pone.0189154.s002.tif]

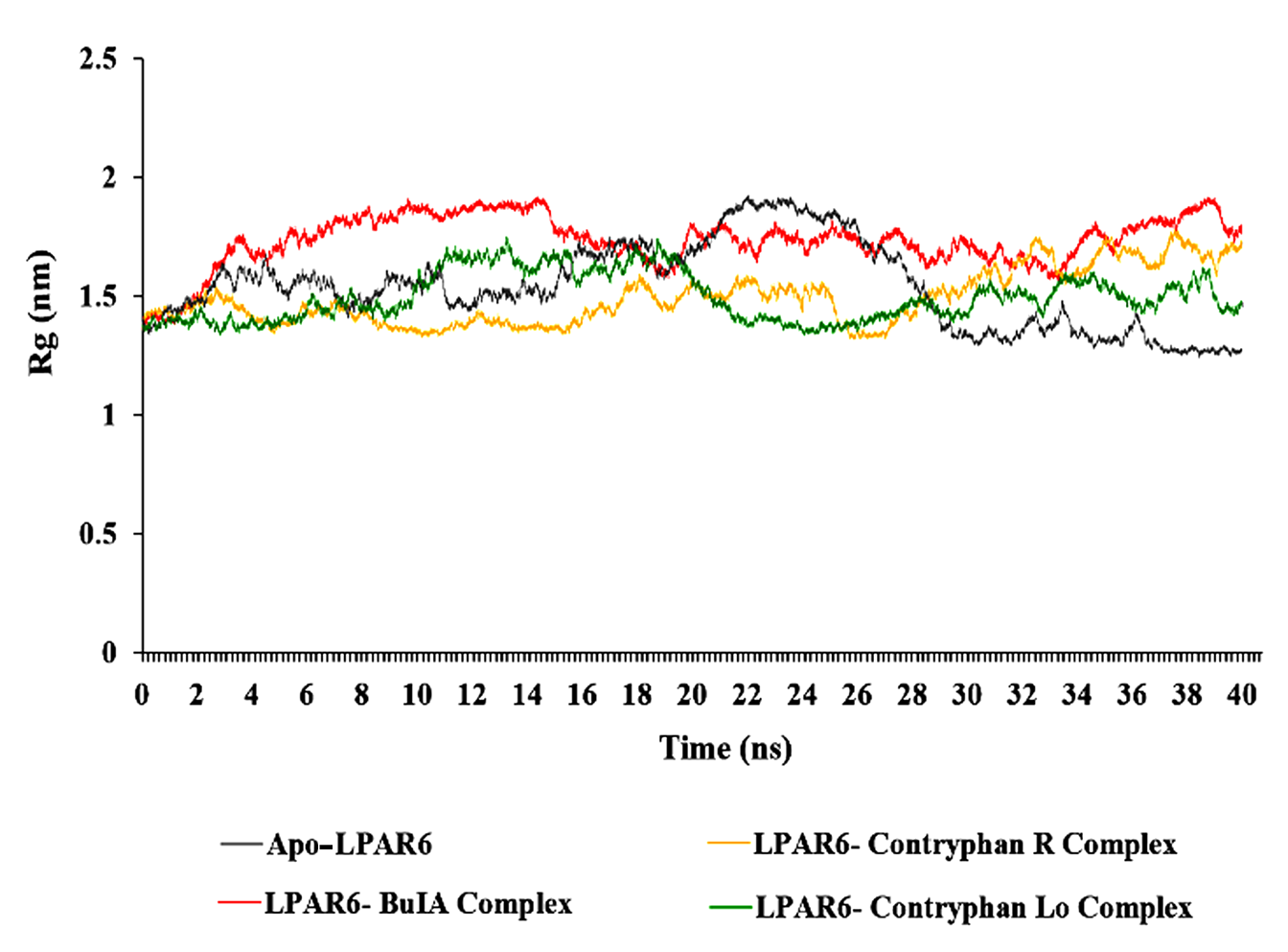

Supplement: S3 Fig — (TIF) [file pone.0189154.s003.tif]

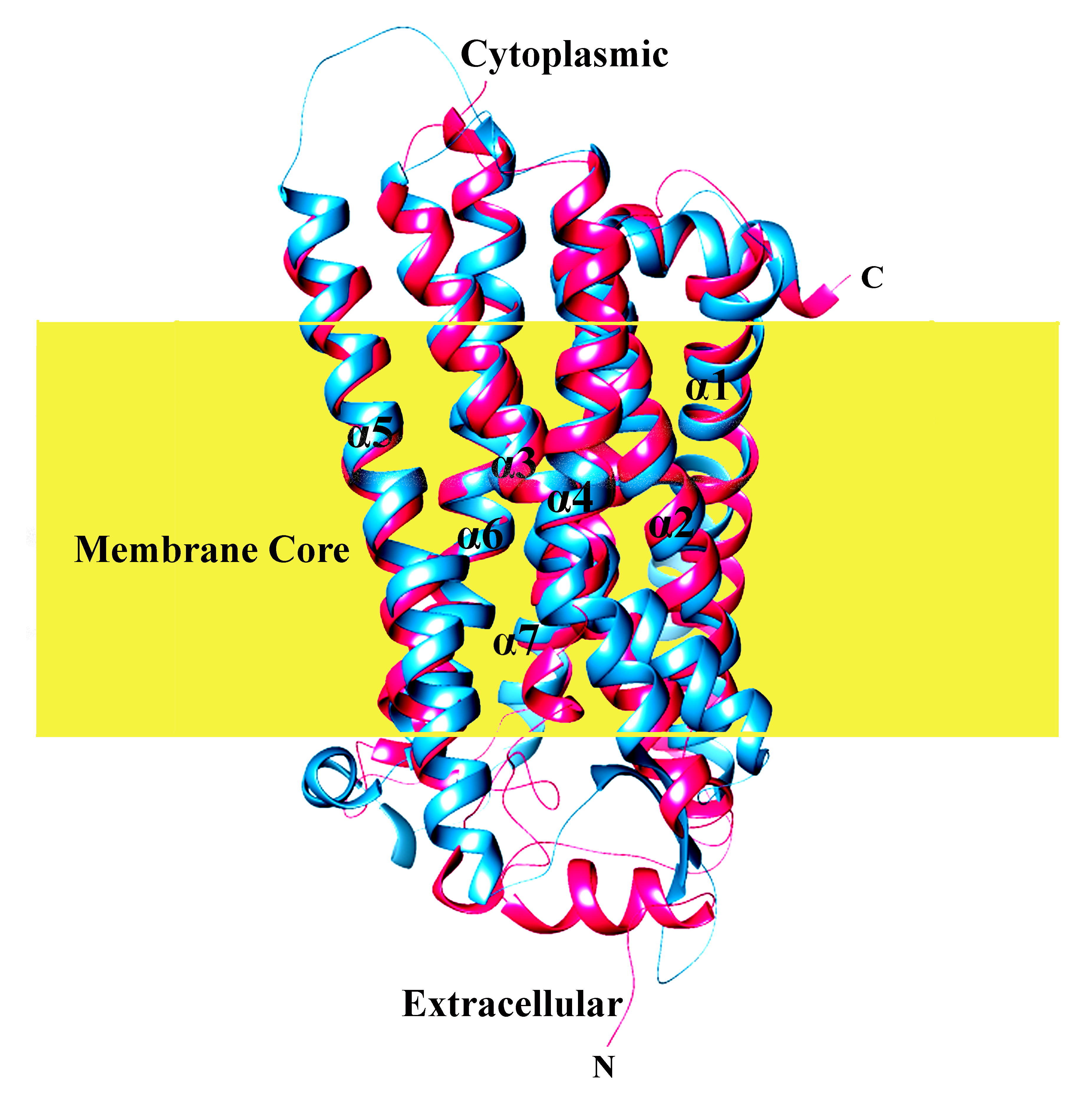

Supplement: S4 Fig — Modelled structure of LPAR6 is superimposed in 3D space with known structure of LPAR1 (PDB ID: 4Z34). LPAR6 is represented in light blue while LPAR1 is depicted in deep pink. Seven aligned transmembrane segments are numbered from 1 to 7 in black color. (TIF) [file pone.0189154.s004.tif]
