# Supplementary material for: Alpha conotoxin-BuIA globular isomer is a competitive antagonist for oleoyl-L-alpha-lysophosphatidic acid binding to LPAR6; A molecular dynamics study
Source: PLoS One. 2017 Dec 6;12(12):e0189154. doi: 10.1371/journal.pone.0189154 (PMC5718415; doi:10.1371/journal.pone.0189154)
Supplement: S1 Table — PDB IDs along with primary sequences and pharmacological classes of considered conotoxins. (DOCX) [file pone.0189154.s005.docx]

**S1 Table: Details of explored conotoxins.** PDB IDs along with primary sequences and pharmacological classes of considered conotoxins.

| Class | Full Names | PDB ID | Protein Sequence |
| --- | --- | --- | --- |
| Alpha | SI | 1QMW | ICCNPACGPKYSCX |
| Alpha | GI [S12>Benzoylphenylalanine] | 2FR9 | ECCNPACGRHYXC |
| Alpha | GI [N4>Benzoylphenylalanine] | 2FRB | ECCXPACGRHYSC |
| Alpha | Vc1.1 | 2H8S | GCCSDPRCNYDHPEICX |
| Alpha | OmIA | 2GCZ | GCCSHPACNVNNPHICGX |
| Alpha | ImI | 1G2G | GCCSDPRCAWRCX |
| Alpha | BuIA | 2I28 | GCCSTPPCAVLYCX |
| Alpha | ImI [R11E] | 1E74 | GCCSDPRCAWECX |
| Alpha | ImI [R7L] | 1E75 | GCCSDPLCAWRCX |
| Alpha | ImI [D5N] | 1E76 | GCCSNPRCAWRCX |
| Alpha | GI | 1NOT | ECCNPACGRHYSCX |
| Alpha | Pni1 | 1PEN | GCCSLPPCAANNPDYCX |
| Alpha | AuIB | 1MXN | GCCSYPPCFATNPDCX |
| Alpha | GID | 1MTQ | IRDECCSNPACRVNNPHVC |
| Alpha | PnIB | 1AKG | GCCSLPPCALSNPDYCX |
| Alpha | SI | 1HJE | ICCNPACGPKYSCX |
| Alpha | EI | 1K64 | RDPCCYHPTCNMSNPQICX |
| Alpha | GI | 1XGC | ECCNPACGRHYSCX |
| Alpha | Epi [sTy15>Y] | 1A0M | GCCSDPRCNMNNPDYCX |
| Alpha | MII | 1M2C | GCCSNPVCHLEHSNLCX |
| Alpha | GI | 1XGA | ECCNPACGRHYSCX |
| Alpha | GI | 1XGB | ECCNPACGRHYSCX |
| Alpha | ImI | 1IMI | GCCSDPRCAWRCX |
| Alpha | ImI | 1CNL | GCCSDPRCAWRCX |
| Alpha | GIC | 1UL2 | GCCSHPACAGNNQHICX |
| Alpha | ImI | 1IM1 | GCCSDPRCAWRCX |
| Alpha | MII | 1MII | GCCSNPVCHLEHSNLCX |
| Alpha | PnIA [A10L,D14K,sTy15Y] | 2BR8 | GCCSLPPCALNNPKYCX |
| Alpha | AuIB | 1DG2 | GCCSYPPCFATNPDCX |
| Alpha | cMII-6 | 2AJW | GCCSNPVCHLEHSNLCGGAAGG |
| Alpha | cMII-7 | 2AK0 | GCCSNPVCHLEHSNLCGAGGAAG |
| Alpha | ImI | 2BYP | GCCSDPRCAWRCX |
| Alpha | ImI | 2C9T | GCCSDPRCAWRCX |
| Alpha | PIA | 1ZLC | RDPCCSNPVCTVHNPQICX |
| Alpha | PIIIE | 1AS5 | HPPCCLYGKCRRYPGCSSASCCQRX |
| Alpha | CnIA | 1B45 | GRCCHPACGKYYSCX |
| Alpha | PIIIE | 1JLO | HPPCCLYGKCRRYPGCSSASCCQRX |
| Alpha | PIIIF | 1JLP | GPPCCLYGSCRPFPGCYNALCCRKX |
| Alpha | PIVA [Hyp7P,Hyp13P] | 1P1P | GCCGSYPNAACHPCSCKDRXSYCGQX |
| Alpha | EIVA | 1PQR | GCCGPYPNAACHPCGCKVGRPPYCDRPSGGX |
| Alpha | ImI [C2U,C8U] | 2BC7 | GCCSDPRCAWRCX |
| Alpha | ImI [C2U,C3U,C8U,C12U] | 2BC8 | GCCSDPRCAWRCX |
| Alpha | ImI deamidated | 2IGU | GCCSDPRCAWRC |
| Alpha | ImI [P6K] | 2IFZ | GCCSDKRCAWRCX |
| Alpha | ImI [P6A] | 2IFI | GCCSDARCAWRCX |
| Alpha | ImI [P6K] deamidated | 2IFJ | GCCSDKRCAWRC |
| Alpha | TxIA | 2UZ6 | GCCSRPPCIANNPDLCX |
| Alpha | BuIA | 2NS3 | GCCSTPPCAVLYCX |
| Alpha | RgIA | 2JUT | GCCSDPRCRYRCR |
| Alpha | BuIA | 4EZ1 | GCCSTPPCAVLYCX |
| Chi | Cyclic MrIA | 2J15 | NGVCCGYKLCHPCAG |
| Chi | MrIB C-term amidated | 1IEO | VGVCCGYKLCHPCX |
| Chi | CMrVIA | 2B5P | VCCGYKLCHPC |
| Chi | CMrVIA | 2B5Q | VCCGYKLCHPC |
| Chi | MrIA | 2EW4 | NGVCCGYKLCHPC |
| Chi | CMrVIA [K6P] | 2IH6 | VCCGYPLCHPC |
| Chi | CMrVIA [K6P] amidated | 2IH7 | VCCGYPLCHPCX |
| Chi | CMrVIA amidated | 2IHA | VCCGYKLCHPCX |
| Delta | TxVIA | 1FU3 | WCKQSGEMCNLLDQNCCDGYCIVLVCT |
| Delta | EVIA | 1G1P | DDCIKXYGFCSLPILKNGLCCSGACVGVCADLX |
| Delta | EVIA | 1G1Z | DDCIKXYGFCSLPILKNGLCCSGACVGVCADLX |
| Delta | Evia Loop 2 Analog | 1SBU | GFASLXILKNG |
| Delta | Am2766 | 1YZ2 | CKQAGESCDIFSQNCCVGTCAFICIEX |
| epsilon | TxVA | 1WCT | ECCEDGWCCTAAX |
| iota | RXIA | 2P4L | GPSFCKADEKPCEYHADCCNCCLSGICAPSTNWILPGCSTSSFFKI |
| Iota | RXIA | 2JTU | GPSFCKADEKPCEYHADCCNCCLSGICAPSTNWILPGCSTSSFFKI |
| kappa | PVIIA | 1KCP | CRIPNQKCFQHLDDCCSRKCNRFNKCVX |
| Kappa | PVIIA | 1AV3 | CRIPNQKCFQHLDDCCSRKCNRFNKCVX |
| Kappa | PlXIVA | 2FQC | FPRPRICNLACRAGIGHKYPFCHCRX |
| mu | conotoxin-GS | 1AG7 | ACSGRGSRCPPQCCMGLRCGRGNPQKCIGAHEDV |
| Mu | GIIIB | 1GIB | RDCCTPPRKCKDRRCKPMKCCAX |
| Mu | SmIIIA | 1Q2J | QRCCNGRRGCSSRWCRDHSRCC |
| Mu | PIIIA | 1R9I | QRLCCGFPKSCRSRQCKPHRCCX |
| Mu | MrVIB | 1RMK | ACSKKWEYCIVPILGFVYCCPGLICGPFVCV |
| Mu | GIIIA | 1TCG | RDCCTPPKKCKDRQCKPQRCCAX |
| Mu | GIIIA [R13A] | 1TCH | RDCCTPPKKCKDAQCKPQRCCAX |
| Mu | GIIIA | 1TCJ | RDCCTPPKKCKDRQCKPQRCCAX |
| Mu | GIIIA [R13A] | 1TCK | RDCCTPPKKCKDAQCKPQRCCAX |
| Mu | KIIIA | 2LXG | CCNCSSKWCRDHSRCCX |
| Mu | CnIIIC | 2YEN | QGCCNGPKGCSSKWCRDHARCCX |
| omega | MVIIC | 1CNN | CKGKGAPCRKTMYDCCSGSCGRRGKCX |
| Omega | MVIIA | 1DW4 | CKGKGAKCSRLMYDCCTGSCRSGKCX |
| Omega | MVIIA | 1DW5 | CKGKGAKCSRLMYDCCTGSCRSGKCX |
| Omega | TxVII | 1F3K | CKQADEPCDVFSLDCCTGICLGVCMW |
| Omega | MVIIA With C-Terminal Gly | 1FEO | CKGKGAKCSRLMYDCCTGSCRSGKCG |
| Omega | SO3 | 1FYG | CKAAGKPCSRIAYNCCTGSCRSGKCX |
| Omega | MVIIA | 1MVI | CKGKGAKCSRLMYDCCTGSCRSGKCX |
| Omega | SVIB | 1MVJ | CKLKGQSCRKTSYDCCSGSCGRSGKCX |
| Omega | GVIA | 1OMC | CKSPGSSCSPTSYNCCRSCNPYTKRCYX |
| Omega | MVIIA | 1OMG | CKGKGAKCSRLMYDCCTGSCRSGKCX |
| Omega | MVIIC | 1OMN | CKGKGAPCRKTMYDCCSGSCGRRGKCX |
| Omega | GVIA [O10>K] | 1TR6 | CKSXGSSCSKTSYNCCRSCNXYTKRCYX |
| Omega | MVIIA[R10K] | 1TT3 | CKGKGAKCSKLMYDCCTGSCRSGKCX |
| Omega | MVIIA | 1TTK | CKGKGAKCSRLMYDCCTGSCRSGKCX |
| Omega | GVIA | 1TTL | CKSPGSSCSPTSYNCCRSCNPYTKRCYX |
| Omega | MVIIC analog | 1V4Q | CKGKGAPCRKTMYDCCKGRCGRRGRCX |
| Omega | GVIA | 2CCO | CKSPGSSCSPTSYNCCRSCNPYTKRCYX |
| Omega | FVIA | 2KM9 | CKGTGKSCSRIAYNCCTGSCRSGKCX |
| Chemically modified | BuIIIB[G2dAla] | 2LOC | VAERCCKNGKRGCGRWCRDHSRCCX |
| Chemically modified | Marmophin [d13>D] | 2JQC | DWEYHAHPKPNSFWT |
| Chemically modified | RgIA [D5E] | 2JUR | GCCSEPRCRYRCR |
| Chemically modified | RgIA [P6V | 2JUQ | GCCSDVRCRYRCR |
| Chemically modified | Vc1.1 [Cys2Agl,Cys8Agl] | 2MFX | GXCSDPRXNYDHPEICX |
| Chemically modified | Vc1.1 [Cys2Agl,Cys8Agl] | 2MFY | GXCSDPRXNYDHPEICX |
| Chemically modified | Vc1.1 [Cys3Agl,Cys16Agl] | 2MG6 | GCXSDPRCNYDHPEIXX |
| Chemically modified | Imi1 | 2MOA | GXASDPRCAWRCX |
| Chemically modified | Cyclic Vc1.1 | 4TTL | GCCSDPRCNYDHPEICGGAAGG |
| Chemically modified | RgIA [Cys2Agl,Cys8Agl] | 2MTO | GXCSDPRXRYRCR |
| Chemically modified | RgIA [Cys3Agl,Cys12Agl] | 2MTT | GCXSDPRCRYRXR |
| Chemically modified | RgIA [Cys3Agl,Cys12Agl | 2MTU | GCXSDPRCRYRXR |
| Chemically modified | Cyclic contryphan | 1D7T | GCXXNPKCX |
| Chemically modified | Contryphan-R [Des-Gly1] | 1DG0 | CPWQPWCX |
| Chemically modified | RXIA [BTr33>W] | 2JRY | GPSFCKADEKPCEYHADCCNCCLSGICAPSTNWILPGCSTSSFFKI |
| Chemically modified | AuIB [ribbon isoform] | 1MXP | GCCSYPPCFATNPDCX |
| Chemically modified | GI antitoxic analog | 1QS3 | CANPACGRHYSX |
| Unclassified | Contryphan-In | 2M6D | GCVLYPWCX |
| Unclassified | Contryphan-In | 2M6E | GCVLYPWCX |
| Unclassified | Contryphan-Sm | 1DFY | GCPWQPWCX |
| Unclassified | Contryphan-Sm | 1DFZ | GCPWQPWCX |
| Unclassified | Conantokin-G | 1AD7 | GEEELQENQELIREKSNX |
| Unclassified | Conantokin-G | 1ONU | GEEELQENQELIREKSNX |
| Unclassified | Conantokin-T | 1ONT | GEEEYQKMLENLREAEVKKNAX |
| Unclassified | Contryphan-Vn | 1NXN | GDCPXKPWCX |
| Unclassified | Conantokin-G | 1AWY | GEEELQENQELIREKSNX |
| Unclassified | Contryphan-R | 1QFB | GCXXEPWCX |
| Unclassified | Conkunitzin-S1 | 1Y62 | KDRPSLCDLPADSGSGTKAEKRIYYNSARKQCLRFDYTGQGGNENNFRRTYDCQRTCLYT |
| Unclassified | TVIIA | 1EYO | SCSGRDSRCPPVCCMGLMCSRGKCVSIYGE |
| Unclassified | GmIXA | 1IXT | SCNNSCQSHSDCASHCICTFRGCGAVNX |
| Unclassified | MrIIIE | 2EFZ | VCCPFGGCHELCYCCDX |
| Unclassified | Conomarphin-Mr1 | 2YYF | DWEYHAHPKPNSFWT |
| Unclassified | Conkunitzin-S2 | 2J6D | ARPKDRPSYCNLPADSGSGTKPEQRIYYNSAKKQCVTFTYNGKGGNGNNFSRTNDCRQTCQYPVG |
| Unclassified | CnVA | 3ZKT | ECCHRQLLCCLRFVX |
| Unclassified | BuIIIB | 2LO9 | VGERCCKNGKRGCGRWCRDHSRCCX |
| Unclassified | CcTx | 4B1Q | APWLVPSQITTCCGYNPGTMCPSCMCTNTC |
| Unclassified | TxIB | 2LZ5 | GCCSDPPCRNKHPDLCX |
| Unclassified | Contryphan-In | 2M6C | GCVLYPWCX |
| Unclassified | Contryphan-In | 2M6F | GCVLYPWCX |
| Unclassified | Contryphan-Lo | 2M6G | GCPWDPWCX |
| Unclassified | Contryphan-Lo | 2M6H | GCPWDPWCX |
| Unclassified | LvIA | 2MDQ | RGCCSHPACNVDHPEICX |
| Unclassified | Lo1a | 2MD6 | EGCCSNPACRTNHPEVCD |
| Unclassified | Ar1446 | 2M61 | CCRLACGLGCHPCCX |
| Unclassified | Ar1248 | 2M62 | GVCCGVSFCYPC |
| Unclassified | TxID | 2M3I | GCCSHPVCSAMSPICX |
