# Supplementary material for: Alpha conotoxin-BuIA globular isomer is a competitive antagonist for oleoyl-L-alpha-lysophosphatidic acid binding to LPAR6; A molecular dynamics study
Source: PLoS One. 2017 Dec 6;12(12):e0189154. doi: 10.1371/journal.pone.0189154 (PMC5718415; doi:10.1371/journal.pone.0189154)
Supplement: S2 Table — Every lowest free energy valued docked complex is observed to record total number of conformation lying in a specific cluster (Clusters) and corresponding energy range of the cluster conformation (Energy Range of Cluster), Average binding energy of the cluster conformations (Binding Energies) and Root mean square deviations between conformations (RMSDs). (DOCX) [file pone.0189154.s006.docx]

Supplementary Table 2: Details of docking clusters in conopeptides-LPAR6 minimum scoring energy complexes. Every lowest free energy valued docked complex is observed to record total number of conformation lying in a specific cluster (Clusters) and corresponding energy range of the cluster conformation (Energy Range of Cluster), Average binding energy of the cluster conformations (Binding Energies) and Root mean square deviations between conformations (RMSDs).

| PDB IDs of Peptides | Clusters | Energy Range of Cluster | Binding Energies | RMSDs |
| --- | --- | --- | --- | --- |
| 1CNL | 30 | 0.47 | -0.35 | 50.31 |
|  | 7 | 0.55 | -0.31 | 44.15 |
|  | 6 | 0.18 | -0.17 | 42.57 |
|  | 5 | 0.31 | -0.02 | 48.36 |
| 1D7T | 7 | 0.28 | -2.55 | 62.54 |
|  | 7 | 0.27 | -1.5 | 57.08 |
| 1DFZ | 6 | 0.48 | -4.57 | 61.77 |
|  | 15 | 0.88 | -3.08 | 64.11 |
|  | 8 | 0.38 | -2.1 | 63.58 |
| 1DG2 | 58 | 0.99 | -2.57 | 72.29 |
|  | 5 | 0.67 | -1.65 | 70.85 |
| 1E74 | 10 | 0.45 | -1.64 | 64.47 |
|  | 8 | 0.25 | -1.59 | 71.88 |
|  | 12 | 0.41 | -1.53 | 52.33 |
|  | 12 | 0.77 | -1.36 | 54.88 |
|  | 8 | 0.5 | -1.03 | 64.51 |
|  | 5 | 0.06 | -0.96 | 58.98 |
|  | 5 | 0.04 | -0.82 | 58.03 |
| 1E75 | 13 | 0.23 | -1.61 | 36.37 |
|  | 6 | 0.15 | -1.36 | 36.95 |
|  | 25 | 0.42 | -1.25 | 41.15 |
|  | 11 | 0.23 | -1.09 | 41.72 |
| 1E76 | 8 | 1.18 | -1.5 | 57.82 |
|  | 11 | 0.7 | -0.69 | 42.97 |
|  | 15 | 0.37 | -0.69 | 43.01 |
| 1G2G | 6 | 0.39 | -0.63 | 67.14 |
|  | 15 | 0.27 | -0.59 | 47.43 |
|  | 8 | 0.53 | -0.13 | 48.62 |
|  | 10 | 0.08 | -0.09 | 47.79 |
|  | 11 | 0.51 | -0.08 | 46.08 |
| 1IM1 | 5 | 0.79 | -0.31 | 43.25 |
|  | 9 | 0.21 | -0.05 | 45.63 |
|  | 10 | 0.45 | -0.05 | 47.78 |
|  | 6 | 0.39 | -0.01 | 53.54 |
| 1IMI_19TH | 19 | 0.34 | -1.23 | 55.06 |
|  | 5 | 0.5 | -1.05 | 51.33 |
|  | 10 | 0.92 | -0.98 | 64.51 |
|  | 14 | 0.55 | -0.89 | 54.9 |
|  | 8 | 0.31 | -0.75 | 58.95 |
|  | 5 | 0.05 | -0.5 | 56.69 |
|  | 6 | 0.07 | -0.25 | 55.52 |
| 1MXN | 47 | 1.13 | -3.3 | 45.21 |
|  | 8 | 0.73 | -2.64 | 45.05 |
|  | 6 | 0.96 | -2.63 | 44.07 |
|  | 8 | 0.12 | -1.98 | 52.56 |
| 1MXP | 8 | 0.47 | -2.65 | 43.16 |
|  | 39 | 0.94 | -2.62 | 48.41 |
|  | 12 | 0.14 | -2.33 | 50.89 |
|  | 14 | 0.29 | -2.22 | 49.98 |
| 1NXN | 7 | 0.49 | -4.53 | 63.77 |
|  | 7 | 0.81 | -1.51 | 44.79 |
|  | 5 | 0.61 | -1.32 | 52.64 |
|  | 5 | 0.2 | -1.21 | 45.23 |
|  | 5 | 0.36 | -1.08 | 61.1 |
| 1QMW | 8 | 0.096 | -0.88 | 68.34 |
|  | 11 | 0.43 | -0.05 | 44.61 |
| 1UL2 | 26 | 0.53 | -0.47 | 51.14 |
|  | 8 | 0.52 | -0.24 | 48.14 |
|  | 6 | 0.41 | -0.18 | 46.4 |
|  | 9 | 0.38 | -0.17 | 44.1 |
| 1A0M_A | 21 | 0.68 | -2.54 | 57.47 |
|  | 5 | 0.05 | -1.9 | 50.88 |
|  | 5 | 0.93 | -1.78 | 51.23 |
|  | 8 | 0.11 | -1.71 | 52.23 |
|  | 6 | 0.13 | -1.7 | 52.89 |
|  | 7 | 0.34 | -1.51 | 53.71 |
|  | 5 | 0.14 | -1.22 | 58.02 |
| 1A0M_B | 24 | 1.3 | -1.29 | 47.75 |
|  | 10 | 0.63 | -0.77 | 46.87 |
|  | 14 | 0.84 | -0.6 | 50.38 |
|  | 12 | 0.2 | -0.15 | 52.02 |
| 1AKG | 6 | 0.5 | -3.72 | 48.14 |
|  | 20 | 1.23 | -3.64 | 47.9 |
|  | 9 | 0.65 | -2.92 | 48.45 |
|  | 7 | 0.12 | -2.48 | 62.83 |
| 1NOT | 6 | 1.07 | -2.66 | 48.75 |
|  | 21 | 0.47 | -2.16 | 57.84 |
|  | 12 | 0.5 | -2.04 | 52.81 |
|  | 10 | 0.46 | -1.83 | 50.98 |
| 1PEN | 26 | 1.12 | -4.13 | 42.99 |
|  | 10 | 0.32 | -3.84 | 41.48 |
|  | 14 | 0.48 | -3.68 | 42.11 |
|  | 9 | 0.98 | -3.61 | 48.65 |
| 2BYP_J | 18 | 1.45 | -2.73 | 108.73 |
|  | 9 | -1.85 | -0.02 | 105.97 |
|  | 18 | 0.38 | -1.77 | 104.34 |
|  | 13 | 0.5 | -1.7 | 107.4 |
|  | 7 | 0.28 | -1.51 | 105.45 |
|  | 7 | 0.3 | -1.43 | 109.38 |
| 2C9T_T | 11 | 0.18 | -2.51 | 72.75 |
|  | 8 | 0.36 | -1.99 | 54.22 |
|  | 7 | 0.52 | -1.87 | 73.38 |
|  | 24 | 0.65 | -1.68 | 53.12 |
| 2UZ6_O | 30 | 0.43 | -1.83 | 98.4 |
|  | 12 | 0.53 | -1.17 | 95.29 |
|  | 11 | 0.19 | -1.03 | 96.55 |
| 2UZ6_P | 10 | 0.21 | -2.41 | 24.63 |
|  | 22 | 0.77 | -1.87 | 38.41 |
|  | 9 | 0.21 | -1.1 | 22.22 |
|  | 10 | 0.73 | -1.01 | 39.09 |
|  | 5 | 0.17 | -0.97 | 40.99 |
| 2UZ6_T | 9 | 0.41 | -1.74 | 59.05 |
|  | 12 | 1.12 | -1.65 | 64.42 |
|  | 6 | 0.31 | -1.3 | 59.81 |
|  | 12 | 0.38 | -1.27 | 64.56 |
|  | 5 | 0.21 | -1.19 | 59.64 |
|  | 8 | 0.43 | -1.1 | 63 |
|  | 12 | 0.34 | -0.84 | 66.81 |
| 4TTL | 6 | 0.34 | -2.13 | 53.77 |
|  | 5 | 0.02 | -2.1 | 59.44 |
|  | 6 | 0.05 | -2.03 | 37.42 |
|  | 7 | 0.12 | -1.98 | 44.58 |
|  | 7 | 0.13 | -1.95 | 56.8 |
|  | 8 | 0.1 | -1.87 | 52.67 |
|  | 7 | 0.55 | -1.74 | 57.35 |
|  | 5 | 0.14 | -1.68 | 47.49 |
| 2MD6 | 10 | 1.14 | -1.21 | 57.49 |
| 2MFY | 11 | 0.79 | -0.04 | 41.54 |
| 1XGA | 17 | 1.11 | -0.73 | 44.3 |
|  | 6 | 0.27 | -0.33 | 64.04 |
|  | 6 | 0.94 | -0.14 | 41.24 |
| 1XGB | 49 | 0.78 | -0.59 | 46.68 |
|  | 9 | 0.34 | -0.09 | 43.19 |
|  | 9 | 0.3 | -0.03 | 42.95 |
| 2MFX | 3 | 0.1 | -0.67 | 25.78 |
| 2AJW | 23 | 0.22 | -1.18 | 53.32 |
|  | 6 | 0.1 | -0.89 | 53.93 |
|  | 16 | 0.73 | -0.89 | 46.36 |
|  | 12 | 0.15 | -0.86 | 43.78 |
|  | 5 | 0.1 | -0.58 | 43.37 |
|  | 8 | 0.31 | -0.48 | 45.39 |
| 2AK0 | 25 | 1.13 | -2.13 | 43.3 |
|  | 10 | 0.61 | -1.65 | 40.94 |
|  | 8 | 0.51 | -1.42 | 39.37 |
|  | 7 | 0.33 | -0.7 | 51.42 |
|  | 8 | 0.3 | -0.64 | 50.32 |
| 2EFZ | 12 | 0.53 | -1.68 | 37.95 |
|  | 23 | 0.21 | -1.5 | 47.39 |
|  | 15 | 0.21 | -1.49 | 59.27 |
|  | 11 | 0.41 | -1.43 | 45.7 |
|  | 5 | 0.09 | -1.2 | 45.63 |
| 2FR9 | 26 | 0.98 | -0.89 | 49.43 |
|  | 12 | 0.54 | -0.27 | 48.65 |
| 2FRB | 25 | 0.63 | -0.02 | 53.64 |
| 2GCZ | 26 | 1.56 | -0.74 | 43.31 |
|  | 13 | 0.52 | -0.72 | 43.18 |
|  | 6 | 0.44 | -0.06 | 44.62 |
| 2I28 | 8 | 0.5 | -2.24 | 48.87 |
|  | 6 | 0.57 | -2.16 | 49.29 |
|  | 12 | 0.48 | -2.07 | 51.14 |
| 2IFI | 27 | 1.34 | -0.43 | 61.94 |
|  | 9 | 0.62 | -0.09 | 62.1 |
| 2M3I | 13 | 0.35 | -0.98 | 48.82 |
|  | 15 | 0.72 | -0.78 | 54.95 |
|  | 6 | 0.3 | -0.56 | 68.08 |
|  | 9 | 0.28 | -0.37 | 55.11 |
|  | 5 | 0.21 | -0.36 | 55.17 |
| 2M6D | 6 | 0.61 | -3.23 | 72.66 |
|  | 5 | 0.36 | -2.67 | 57.65 |
|  | 5 | 0.19 | -2.63 | 46.74 |
|  | 6 | 0.7 | -2.55 | 55.88 |
|  | 11 | 0.21 | -2.49 | 62.32 |
|  | 4 | 0.3 | -2.45 | 55.33 |
| 2M61 | 15 | 0.76 | -3.22 | 48.22 |
|  | 7 | 0.81 | -2.58 | 68.96 |
|  | 24 | 1.18 | -2.46 | 55.95 |
|  | 19 | 0.46 | -2.24 | 56.82 |
| 2M62 | 8 | 0.59 | -2.02 | 48.16 |
|  | 14 | 0.56 | -1.6 | 54.47 |
|  | 6 | 0.11 | -1.58 | 54.92 |
|  | 7 | 0.21 | -1.54 | 54.26 |
|  | 9 | 0.19 | -1.49 | 51.85 |
|  | 6 | 0.09 | -1.28 | 49.64 |
| 2MDQ | 8 | 0.12 | -0.98 | 51.82 |
|  | 39 | 0.53 | -0.96 | 54.17 |
|  | 13 | 0.11 | -0.77 | 50.07 |
|  | 6 | 0.15 | -0.52 | 52.95 |
| 2MOA | 10 | 0.1 | -0.13 | 56.78 |
|  | 11 | 0.59 | -0.02 | 57.83 |
|  | 9 | 0.31 | -0.01 | 70.37 |
| 2NS3 | 34 | 0.75 | -3.11 | 48.04 |
|  | 5 | 0.16 | -2.06 | 52.68 |
|  | 7 | 0.16 | -1.92 | 54.68 |
| 2B5P | 8 | 0.04 | -1 | 76.75 |
|  | 5 | 0.16 | -0.85 | 34.77 |
|  | 14 | 0.15 | -0.83 | 59.47 |
|  | 37 | 0.06 | -0.46 | 59.25 |
|  | 7 | 0.15 | -0.36 | 59.01 |
|  | 5 | 0.02 | -0.31 | 59.7 |
| 2B5Q | 77 | 0.02 | -1.42 | 59.01 |
|  | 5 | 0 | -1.33 | 49.1 |
|  | 8 | 0.02 | -0.98 | 64.06 |
| 2IH7 | 74 | 0.26 | -2.78 | 64.56 |
|  | 9 | 0.03 | -2.63 | 57.29 |
| 2IHA | 13 | 0.03 | -1.4 | 66.48 |
|  | 59 | 0.05 | -1.37 | 53.52 |
|  | 9 | 0.03 | -0.72 | 58.25 |
|  | 7 | 0.02 | -0.71 | 61.63 |
| 2J15 | 25 | 0.17 | -2.39 | 40.67 |
|  | 54 | 0.05 | -1.79 | 49.96 |
|  | 5 | 0.03 | -1.67 | 43.5 |
|  | 9 | 0.03 | -1.66 | 42.26 |
| 1DFY | 5 | 2.11 | -6.34 | 61.58 |
|  | 5 | 0.75 | -4.49 | 60.79 |
|  | 9 | 0.64 | -2.29 | 64.23 |
|  | 14 | 0.23 | -2.05 | 60.12 |
|  | 8 | 0.08 | -1.46 | 63.13 |
| 2IH6 | 5 | 0.01 | -3.96 | 76.44 |
|  | 8 | 0.04 | -2.99 | 50.6 |
|  | 54 | 0.13 | -2.7 | 61.39 |
|  | 8 | 0.06 | -2.69 | 60.99 |
|  | 5 | 0.02 | -2.37 | 62.9 |
| 2M6E | 11 | 0.35 | -5.18 | 71.17 |
|  | 28 | 1.04 | -4.26 | 53.43 |
|  | 13 | 0.15 | -3.51 | 50.74 |
|  | 9 | 0.46 | -3.23 | 55.37 |
| 2M6F | 5 | 0.43 | -5.9 | 72.96 |
|  | 6 | 1.05 | -5.18 | 70.96 |
|  | 13 | 0.34 | -2.68 | 55.1 |
|  | 8 | 0.29 | -2.6 | 65.72 |
|  | 8 | 0.38 | -2.47 | 57.06 |
| 2M6G | 19 | 0.6 | -4.5 | 53.21 |
|  | 16 | 0.78 | -4.26 | 55.57 |
|  | 5 | 0.18 | -4.05 | 42.16 |
| 2M6H | 7 | 0.94 | -6.46 | 70.78 |
|  | 15 | 0.71 | -6.16 | 72.67 |
|  | 14 | 1.1 | -4.65 | 54.15 |
|  | 5 | 0.11 | -3.88 | 52.56 |
|  | 5 | 0.29 | -3.74 | 54.7 |
| 2M6C | 8 | 0.65 | -4.96 | 69.93 |
|  | 39 | 0.77 | -4.08 | 52.79 |
|  | 7 | 0.44 | -2.87 | 58.21 |
| 4EZ1_K | 23 | 0.79 | -4.14 | 35.75 |
|  | 10 | 0.29 | -3.81 | 33.12 |
|  | 5 | 0.48 | -3.77 | 36.47 |
|  | 6 | 0.4 | -3.61 | 36.07 |
|  | 6 | 0.28 | -3.39 | 37.69 |
|  | 4 | 1.65 | -7.72 | 58.53 |
| 1QFB | 11 | 0.85 | -5.22 | 63.07 |
|  | 6 | 0.26 | -2.57 | 64.62 |
|  | 9 | 0.2 | -2.42 | 59.24 |
|  | 5 | 0.53 | -2.41 | 64.82 |
|  | 7 | 0.37 | -2.31 | 62.32 |
| 1DG0 | 8 | 0.03 | -7.7 | 62.97 |
|  | 7 | 0.16 | -5.77 | 63.81 |
|  | 6 | 1.18 | -5.66 | 63.32 |
|  | 5 | 0.01 | -3.76 | 54.44 |
|  | 5 | 0.28 | -3.41 | 67.77 |
