# Supplementary material for: Alpha conotoxin-BuIA globular isomer is a competitive antagonist for oleoyl-L-alpha-lysophosphatidic acid binding to LPAR6; A molecular dynamics study
Source: PLoS One. 2017 Dec 6;12(12):e0189154. doi: 10.1371/journal.pone.0189154 (PMC5718415; doi:10.1371/journal.pone.0189154)
Supplement: S3 Table — (DOCX) [file pone.0189154.s007.docx]

S3 Table: Comparison of drug-like properties of selected conopeptides.

| Parameter | 4EZ1 | 1QFB | 1DG0 | 2M6G | 2M6H | 1DFY | 2M6C | 2M6E | 2IH6 |
| --- | --- | --- | --- | --- | --- | --- | --- | --- | --- |
| Mol. Wt. | 1316.59 | 977.12 | 920.07 | 963.09 | 1074.42 | 976.14 | 1051.47 | 940.14 | 1225.52 |
| Theoretical Iso electric point | 5.5 | 4 | 4 | 3.8 | 3.8 | 5.51 | 5.51 | 5.51 | 7.86 |
| Extinction coefficient, assuming all pairs of Cys residues form cysteines | 1740 | 11125 | 11125 | 11125 | 11125 | 11125 | 7115 | 7115 | 1740 |
| Extinction coefficient, assuming all Cys residues are reduced | 1490 | 11000 | 11000 | 11000 | 11000 | 11000 | 6990 | 6990 | 1490 |
| Estimated half-life in hours (mammalian reticulocytes, in vitro) | 30 | 30 | 1.2 | 30 | 30 | 30 | 30 | 30 | 100 |
| Estimated half-life in hours (yeast, in vivo) | 20 | 20 | 20 | 20 | 20 | 20 | 20 | 20 | 20 |
| Estimated half-life in hours (Escherichia coli, in vivo) | 10 | 10 | 10 | 10 | 10 | 10 | 10 | 10 | 10 |
| Grand average of hydropathicity (GRAVY) | 1.031 | -0.487 | -0.5 | -0.487 | -0.433 | -0.487 | 0.978 | 1.1 | 0.691 |
| Instability index | 18.25 | 49.7 | 55.37 | 25.63 | 22.78 | 49.7 | 9.91 | 11.15 | 13.82 |
