# Supplementary material for: Alpha conotoxin-BuIA globular isomer is a competitive antagonist for oleoyl-L-alpha-lysophosphatidic acid binding to LPAR6; A molecular dynamics study
Source: PLoS One. 2017 Dec 6;12(12):e0189154. doi: 10.1371/journal.pone.0189154 (PMC5718415; doi:10.1371/journal.pone.0189154)
Supplement: S4 Table — (DOCX) [file pone.0189154.s008.docx]

| **PDB IDs** | **Binding Energies (Kcal/mol)** | **LPAR6 residues implicated in developing associations** |
| --- | --- | --- |
| 4EZ1_K | -7.72 | Tyr76, Phe77, Arg80, Trp82, Phe96, **Tyr97,** Gln160, **Ser165,** Cys168, Phe169, Leu181, Leu249, Tyr252, **Arg270,** Tyr273, Pro274, Leu277 |
| 1DG0 | -7.7 | Arg73, Tyr76, Phe77, Arg80, Phe96, Tyr97, Cys168, Phe169, Glu170, Leu181, Val185, Tyr245, Leu249, **Tyr252,** Arg270, Tyr273, Leu277 |
| 2M6G | -6.73 | Thr69, Arg73, Arg80, Trp82, Ser92, Val93, Phe96, Phe169, Glu166, Ala167, **Cys168, Tyr245,** Arg270, Tyr273 |
| 2M6H | -6.46 | Thr69, Arg73, Phe77, Ser92, Val93, Phe96, Tyr252, Arg270, Tyr273, Pro274, Leu277 |
| 1DFY | -6.34 | Tyr76, Phe77, Arg80, Asn81, Trp82, Gln160, Glu166, Cys168, Phe169, Glu170, Trp177, Leu181, Tyr273, Arg270, Tyr252 |
| 2M6F | -5.9 | Arg73, Tyr76, Phe77, Arg80, Trp82, Val93, Gln160, **Cys168,** Phe169, Tyr273, Arg270 |
| 1QFB | -5.82 | Arg73, Tyr76, Phe77, Asn81, Arg80, Trp82, Val93, Phe96, Gln155, **Glu166,** Ala167, **Cys168,** Phe169, Leu181, Tyr245, Leu249, Arg270, Leu277, **Tyr273,** Pro274 |
| 2M6E | -5.18 | Arg73, Tyr76, Phe77, Tyr97, Cys168, PHe169, Glu170, Trp177, Tyr273, Pro274, Leu277 |
| 2M6C | -4.96 | Tyr76, Arg80, Asn81, Trp82, Val93, Tyr97, Cys168, Gln160, Phe169, Trp177, Lys178, Leu181, Tyr252, Tyr273 |
| 1AKG | -4.63 | Tyr55, Leu105, Thr108, Cys109, Val112, **Asp113,** Leu116, **Lys123,** Arg128, **Asn132,** Ile135, Val136, Thr143, Leu199, |
| 2M6D | -4.58 | Arg73, Tyr76, Phe77, Trp82, Val93, **Gln155,** Ala167, Cys168, Phe169, Glu170, Trp177, Leu181, Arg270, Tyr273, |
| 1NXN | -4.53 | Thr69, Tyr76, Phe77, Arg80, Asn81, Trp82, Cys168, Leu249, Tyr252, Arg270, Tyr273 |
| 2IH6 | -4.3 | Arg80, Ala167, **Gln160,** Cys168, Phe169, Trp177, Lys178, Leu181, Tyr252, Tyr273 |
| 1PEN | -4.13 | Lys229, Phe232, Val233, Ile237, Phe240, Phe286, Ile289, Val290, Phe293, Thr294 |
| 2NS3 | -3.91 | Arg73, Phe77, Arg80, **Gln160,** Ala167, Cys168, Phe169, Trp177, Leu181, Tyr245, Tyr252, Tyr273, Pro274, |
| 2I28 | -3.51 | Tyr76, Phe77, Gln160, Ala167, Cys168, Phe169, **Glu170,** Phe172, Leu181, Tyr252, **Arg270,** Tyr273 |
| 1MXN | -3.3 | Lys229, Val233, Ile237, Phe286, Ile289, Phe293, Thr294 |
| 2C9T_T | -3.25 | Tyr273, Tyr76, Phe77, Arg80, Asn81, Trp82, Gln160, Asn163, Ser165, Trp177, Tyr252, **Arg270** |
| 2M61 | -3.22 | **Asp296, Lys226**  Ile237, Phe293, Thr294, Lys229, Phe232, Val233, Ile236, Val290 |
| 1NOT | -2.94 | **Phe77, Tyr76**  Arg80, Arg270, Cys168, Leu249, Ala167, Tyr273, Gln160, Tyr245, Tyr252, Trp177, Leu181 |
| 4TTL | -2.9 | **Lys123, Arg128, Cys109**  Asp113, Val112, Val140, Leu105, Thr108, Val136, Asn132, Ile135 |
| 2IH7 | -2.8 | **Trp177, Pro173,**  Ala175, Gln160, Glu174, Gly161, Phe169, His158, Phe172 |
| 2BYP_J | -2.73 | Phe84, Phe68, Pro71, Leu32, Phe24, Phe28 |
| 1MXP | -2.66 | **Val136**  Pro196, Ile195, Gly146, Tyr101, Val191, Thr143, Phe187, Gly139, Val140 |
| 2M62 | -2.64 | **Asn132, Cys109**  Val191, Ile135, Ile195, Leu105, Thr143, Val136, Tyr55, Val112, Asp113 |
| 1DG2 | -2.57 | Phe293, Ile237, Val290, Ile289, Phhe240, Phe286, Cys241, Ile279, Ser282 |
| 1D7T | -2.55 | Tyr97, Glu170, Phe169, Trp82, Val93, Cys168, Tyr76, Phe77, Arg80, Arg270, Tyr273, Ala167, Phe169 |
| 1A0M_A | -2.54 | **Lys229**  Phe84, Phe68, Pro71, Leu32, Phe24, Phe28 |
| 2UZ6_P | -2.41 | Phe75, Leu88, Phe72, Phe84, Pro83, Pro71, Phe24, Phe28 |
| 2J15 | -2.39 | **Phe293, Thr294, Arg270**  Leu208, Phe232, Val233, Ile236, Val290, Lys226, Met230, Lys229 |
| 2AK0 | -2.13 | Lys229, Ile236, Val233, Val290, Phe293, Thr294, Phe286, Ile237, Cys241, Phe240 |
| 1G2G | -1.86 | **Gln160**  Trp177, Tyr245, Tyr252, Trp82, Cys168, Tyr97, Leu181, Ala167, Arg80, Leu249, Pro274, Tyr273, Arg73, Phe77 |
| 2UZ6_O | -1.83 | Phe293, Asp296, Lys229, Thr294, Val233, Phe232, Ile289, Ile236, Cys241, Phe240, Ile237, Phe286 |
| 1E74 | -1.76 | Thr138, Trp141, Met61, Ile145, Leu65, Met94, Leu95, Phe68, Ile91, Leu88 |
| 2UZ6_T | -1.74 | Val29, Phe72, Leu32, Phe84, Leu88, Phe68, Phe28, Ile33, Phe24, Pro71 |
| 2EFZ | -1.68 | Leu211, Leu208, Lys229, Val201, Phe232, Val233, Ile236, Phe240, Ile237 |
| 1E75 | -1.61 | Ile236, Ile237, Val290, Val233, Thr294, Phe293, Lys226, Phe286 |
| 1E76 | -1.5 | Ile33, Phe84, Val29, Phe68, Pro71, Phe75, Leu32, Ile74, Phe28, Phe24 |
| 2B5Q | -1.42 | Ile289, Met23, Leu30, Val27, Met26, Phe286, Ile279, Ser282, Cys241, Ile237, Phe240 |
| 2IHA | -1.4 | Phe84, Phe68, Phe28, Phe24, Phe75, Pro71, Phe75, Ile74 |
| 1A0M_B | -1.29 | **Ser25**  Phe240, Phe286, Ile289, Ile237, Lys229, Ile236, Phe232, Val233 |
| 1IM1 | -1.25 | **Glu170**  Gln160, Gly161, Trp177, Glu174, Ala175, Asn171, Phe172, Leu181, Phe169 |
| 1IMI_19TH | -1.25 | **His158, Arg270**  Ser165, Phe77, Ala167, Phe96, Gln160, Leu249, Tyr97, Leu181, Val185, Phe169, Ser92, Cys168, Arg80, Trp82 |
| 2AJW | -1.18 | Ile237, Phe286, Leu19, Val27, Met23, Ile279, Cys22, Met26 |
| 2M3I | -1.08 | Phe240, Phe293, Ile236, Ile237, Val233, Phe232, Lys229, Phe286, Ile289 |
| 2B5P | -1 | **Asn163**  Gln160, Arg270, Trp82, Tyr76, Ala167, Arg80, Phe169, Cys168, Tyr273 |
| 2MDQ | -0.98 | Ile237, Phe240, Ile236, Val233, Phe286, Leu30, Ile289, Phe293, Val290 |
| 2FR9 | -0.89 | Phe240, Ile236, Phe293, Phe286, Val290, Ile237, Val233, Lys229, Phe232, Leu208 |
| 1QMW | -0.88 | Phe28, Tyr17, Thr78, Pro71, Ile74, Phe84, Phe72, Phe75 |
| 2GCZ | -0.74 | Ile237, Lys229, Ile236, Phe293, Thr294, Phe240, Val233, Phe286, Val290, Ile289 |
| 1XGA | -0.73 | **Thr294, Lys229**  Ile237, Val233, Ile236 |
| 1UL2 | -0.69 | **Asn306, Trp307, Lys305, Lys47**  Met304, Tyr40, Val45, Cys44 |
| 2MFX | -0.67 | **Lys221**  Lys125, Lys130, Thr126, Leu46, Leu127, Lys47 |
| 1XGB | -0.59 | **Phe293**  Ile289, Ile237, Val233, Val290, Thr294, Phe286 |
| 2IFI | -0.43 | Ile237, Ile236, Phe293, Val290, Val233, Phe286, Phe240, Ile279 |
| 1CNL | -0.35 | Phe293, Phe286, Ile237, Phe240 |
| 2MOA | -0.28 | Phe75, Tyr17, Phe84, Ile74, Phe24, Ser25, Pro71, Phe72 |
| 2MFY | -0.04 | **Lys229**  Thr294, Phe232, Ile222, Val233, Val290, Lys226 |
| 2FRB | -0.03 | **Glu174, Trp177, Lys178**  Phe169, Gly161, Gln160, Ala175 |
